# Chemotherapy and targeted therapy for advanced biliary tract cancers: an umbrella review

**DOI:** 10.1186/s12885-023-10679-8

**Published:** 2023-04-25

**Authors:** Yaoqun Wang, Ningyuan Wen, Shaofeng Wang, Guilin Nie, Yuan Tian, Jiong Lu, Bei Li

**Affiliations:** 1 Division of Biliary Surgery, Department of General Surgery, West China Hospital, Sichuan University, Sichuan, 610041 Chengdu China; 2 Research Center for Biliary Diseases, West China Hospital, Sichuan University, Sichuan, 610041 Chengdu China

**Keywords:** Biliary tract cancer, Chemotherapy, Targeted therapy, Umbrella review, GRADE

## Abstract

**Background:**

Malignant tumors of the biliary system are characterized by a high degree of malignancy and strong invasiveness, and they are usually diagnosed at late stages with poor prognosis. For patients with advanced biliary tract cancer, chemotherapy and targeted therapy are two of the options available to improve prognosis and delay tumor progression. This study aimed to comprehensively evaluate the safety and effectiveness of various chemotherapy schemes for the treatment of advanced biliary tract cancer in published systematic reviews and meta-analyses (SRoMAs).

**Methods:**

An umbrella review method was adopted, which aims to summarize the existing evidence from multiple studies around a research topic. SRoMAs up to April 9, 2022, were identified using PubMed, Web of Science, the Cochrane database, and manual screening. Eligible studies were screened according to inclusion and exclusion criteria. This study had been registered at PROSPERO (CRD42022324548). For each eligible study, we extracted the data of general characteristics and the main findings. The methodological quality of the included studies were assessed by the AMSTAR2 scale, and the quality of evidence was evaluated by the GRADE tools.

**Results:**

A total of 1833 articles were searched; 14 unique articles with 94 outcomes were identified by eligibility criteria. The incidence of skin rash (RR = 18.11, 95% CI 5.13–63.91, GRADE: Moderate) and diarrhea (RR = 2.48, 95% CI 1.2–5.10, GRADE: Moderate) was higher in patients receiving gemcitabine-based chemotherapy plus targeted therapy than in patients receiving gemcitabine monotherapy. The number of patients receiving gemcitabine-based chemotherapy who developed leukopenia (OR = 7.17, 95% CI 1.43–36.08, GRADE: Moderate), anemia (OR = 7.04, 95% CI 2.59–19.12, GRADE: High), thrombocytopenia (RR = 2.45, 95% CI 1.39–4.32, GRADE: Moderate), and neutropenia (RR = 3.30, 95% CI 1.04–10.50, GRADE: Moderate) was significantly higher than that of patients who received gemcitabine-free regimens. In addition, patients receiving S-1 monotherapy had significantly better ORR (RR = 2.46, 95% CI 1.27–4.57, GRADE: Moderate) than patients receiving S-1 + gemcitabine. Patients receiving fluoropyrimidine-based chemotherapy had longer OS (HR = 0.83, 95% CI 0.7–0.99, GRADE: Moderate), higher DCR (0R = 5.18, 95% CI 3.3–10.23, GRADE: Moderate), and higher ORR (0R = 3.24, 95% CI 1.18–8.92, GRADE: Moderate) compared with patients who received 5-FU/LV monotherapy or supportive therapy. Surprisingly, we found evidence that gemcitabine-based chemotherapy did not improve postoperative patients’ OS (HR = 0.91, 95% CI 0.74–1.12, GRADE: Moderate) when compared with best supportive care.

**Conclusions:**

This study comprehensively evaluated the safety and efficacy of chemotherapy or targeted therapy regimens for advanced biliary tract cancer and found 11 outcomes with “Moderate” or “High” levels; however, most of the outcomes were still at “low” or “very low” levels. More randomized controlled studies are needed in the future to further summarize high levels of evidence.

**Supplementary Information:**

The online version contains supplementary material available at 10.1186/s12885-023-10679-8.

## Introduction

### Description of the condition

Biliary tract cancer is relatively rare but highly malignant, which can be divided into gallbladder cancer and cholangiocarcinoma according to different origins. Cholangiocarcinoma can be divided into intrahepatic cholangiocarcinoma, hilar cholangiocarcinoma, and distal cholangiocarcinoma according to different anatomical sites. In most developed countries, the incidence of biliary tract cancer is low (0.35–2 cases per 100,000 people per year). However, in China and other developing countries in East Asia, the incidence of biliary malignancy is much higher than that of developed countries, which is a health problem that cannot be ignored [1]. Biliary tract cancers usually have an insidious onset, and early symptoms are not obvious. The typical symptoms are mainly caused by biliary obstruction and tumor metastasis. About 70% of patients with cholangiocarcinoma were already in advanced stage when diagnosed, and about half of patients with gallbladder cancer were accidentally discovered during or after cholecystectomy [2]. In general, the prognosis of biliary duct cancer is poor, and the 5-year survival rate of patients is only 5%–15% [3]. Currently, although surgical resection or percutaneous radiofrequency ablation is a possible cure for patients with biliary duct cancer [4], only about 20% of patients can receive radical resection due to late diagnosis [5]. Even after surgical treatment, about 50% of patients still have postoperative recurrence [6]. For most nonoperative and postoperative patients with advanced or metastatic biliary tract cancer, chemotherapy or targeted therapy is an option to delay tumor progression and improve prognosis.

### Description of the interventions

In recent years, many clinical trials of chemotherapy have been conducted for biliary carcinoma. For postoperative adjuvant chemotherapy, oral capecitabine (Cap) is recommended [7]. For patients with advanced biliary tract cancer and without targetable alterations, gemcitabine + cisplatin (GP) in the first-line setting remains the standard treatment [8]. In addition, gemcitabine + S-1 (G + S-1) or capecitabine + oxaliplatin (XELOX) is recommended for first-line chemotherapy [9–11], whereas oxaliplatin + 5-FU (mFOLFOX) is recommended for second-line chemotherapy [12]. Research on targeted drugs for biliary duct cancer also achieved preliminary results, and some clinical trials of chemotherapy combined with targeted drugs have been carried out [13, 14]. In terms of immunotherapy, anti-PD-L1 inhibitors such as durvalumab are also being used for clinical treatment [15].

### Why it is important to do this overview

Given the high heterogeneity of biliary tract tumors, significant differences exist in treatment efficacy, toxicity, and prognosis among patients. Many meta-analyses comparing chemotherapy regimens for biliary tract cancer have been published in recent years to determine the best treatment option. These meta-analyses mainly focused on the controversial topics of systematic treatment of biliary tract cancer, such as whether the addition of targeted therapy in the chemotherapy scheme can benefit patients [16, 17], what are the differences in the clinical efficacy of single-drug chemotherapy or combination chemotherapy [18], and the safety comparison between different chemotherapy schemes [19]. However, the quality of evidence in previous studies is uneven, which makes clinical decision-making challenging [20]. In addition, the conclusions of some reviews are inconsistent or contradicting [21, 22], which brings confusion to clinical decision-making. Therefore, an umbrella review is required to summarize and evaluate the relevant evidence-based practices in this area.

Umbrella review is a comprehensive review of existing systematic reviews and meta-analyses in a certain field, aiming at methodological evaluation and quality grade classification of evidence-based research in this field and providing higher-level evidence support for clinical decision making [23, 24]. To date, no umbrella review of evidence-based research related to chemotherapy and targeted therapy regimens for biliary tumors has been published. Therefore, we conducted this study to comprehensively evaluate the safety and efficacy of various chemotherapy and targeted therapy regimens for patients with advanced biliary duct cancer.

## Methods

### Protocol and registration

To evaluate the efficacy and safety of different treatment options for biliary tract cancer, we conducted an umbrella review in this field. The protocol of this study has been registered on PROSPERO at CRD42022324548. This study was reported according to the PRISMA-ScR checklist. Table S1 provides a checklist of specific items of this study.

### Criteria for considering reviews for inclusion

We included all systematic reviews and meta-analyses that met the following criteria in clinical trials (prospective studies and retrospective studies) to evaluate the efficacy and safety of chemotherapy or targeted therapy for patients with biliary tract cancer.

If there was an overlap between the two reviews and the conclusions of both were consistent, that is, multiple reviews contained evidence relevant to the same comparison under the same conditions, we compared each review with the latest review to determine whether the old review contained any clinical research data that had not been included or adequately reported in the recent review. If this was not the case, we did not consider the earlier review. If the final conclusions of the overlapping reviews were inconsistent, we included both to further compare the level of evidence quality between the two to determine the most credible evidence.

#### Inclusion and exclusion criteria

Inclusion criteria:

(1) SRoMAs of prospective or retrospective studies following the PRISMA guidelines; (2) a comparison was made between different chemotherapy or targeted therapy regimens for biliary duct cancer; (3) odds ratio (OR), relative ratio (RR), OR risk ratio (HR), and their corresponding 95% confidence intervals (CIs) among different treatment regimens were summarized and reported; (4) number of original studies included in SRoMAs ≥2; and (5) no restrictions on language types.

Exclusion criteria:

(1)non-SRoMAs; (2) chemotherapy and targeted therapy for other cancers; (3) radiotherapy or local regional treatment for biliary carcinoma; (4) non-human subjects; (5) OR, RR, HR, and their corresponding 95% CIs among different treatment regimens were not summarized or reported; (6) only chemotherapy or no chemotherapy and chemotherapy or surgery were compared, and there was no comparison between chemotherapy regimens; (8) chemotherapy and targeted therapy for many types of cancer; (9) number of studies included in the SRoMAs <2; and (10) low-quality studies with overlapping content and conclusions.

#### Types of participants

Adults 18 years or older were described as having advanced biliary cancer and meeting the indications for chemotherapy or targeted treatment.

#### Types of intervention

We included all chemotherapy or targeted treatment schemes for biliary tract cancer that met clinical standards.

Here, we list the types of comparison schemes included in this study.

- (1)One combination chemotherapy versus another;
- (2)chemotherapy + targeted therapy versus chemotherapy;
- (3)combined chemotherapy versus single-drug chemotherapy;
- (4) observation versus chemotherapy or targeted therapy.

#### Types of outcome measure

##### Primary outcomes

- (1) Indicators related to prognosis: overall survival (OS), disease-free survival (DFS), and progression-free survival (PFS).
- (2) Efficacy-related indicators: disease control rate (DCR), disease response rate (DRR), and overall response rate (ORR).

##### Secondary outcomes

Incidence and nature of adverse effects or toxic effects.

### Search methods for the identification of reviews

Two authors of this study (Yaoqun Wang and Ningyuan Wen) independently conducted a systematic and comprehensive literature search using PubMed, Web Of Science, and the Cochrane Database Of Systematic Reviews. We searched SRoMAs related to chemotherapy and targeted therapies for biliary tract cancer up to April 09, 2022. The following terms/keywords were used in this search strategy: (gallbladder cancer or biliary tract cancer) AND (systematic review or meta-analysis). In addition, we searched the references included in the study, relevant literature from clinical trials or study registration platforms, and gray literature. All differences were resolved through consultation between the authors. Detailed retrieval strategies for this study and manual retrieval of the included literature are shown in Table S2.

### Data collection and analysis

#### Data extraction and management

Two authors (Yaoqun Wang and Ningyuan Wen) independently extracted the data from the included literature. Any discrepancies were.resolved in consultation with a third author (Shaofeng Wang). For each of the included reviews, we extracted the data on basic characteristics, outcomes, and bias estimation.

Data on basic characteristics included: (1) first author; (2) date of publication; (3) original article retrieval time; (4) journal; (5) total number of included studies; (6) type of study; (7) study design; (8) type of chemotherapy; (9) number of studies included in subgroup analysis; (10) interventions and number of cases; (11) control measures and number of cases; and (12) total number of cases included in the meta-analysis.

Data of outcomes: (1) clinical outcomes; (2) effect models used in meta-analysis; (3) estimated effect values (HR, OR, and RR) and 95% CI; (4) P-value of effect value; and (5) heterogeneity (I^2^).

Bias estimation: (1) Egger’s p value and (2) quality assessment tool of meta-analyses (e.g., Cochrane ROB Tool, Jadad Scale, or NOS).

#### Assessment of methodological quality

Methodological and evidence levels were evaluated for each SRoMA included in this umbrella review. AMSTAR2 is a quality evaluation tool used for assessing the methodological quality of randomized or non-randomized preventive and curative studies [25]. It comprises 16 quality criteria involving the whole process of evaluation, such as topic selection, design, registration, data extraction, data statistical analysis, and discussion. In this study, we used AMSTAR2 to evaluate the included reviews and classified them into four grades (High, Moderate, Low, and Critically low) according to their methodological quality. The details of the AMSTAR2 scale are shown in Table 1. Among these 16 items, items 2, 4, 7, 9, 11, 13, and 15 are critical items. The detailed assessment criteria of the AMSTAR2 scale for SRoMAs are as follows [25].

- High: No or one non-critical weakness: the systematic review provided an accurate and comprehensive summary of the results of the available studies that addressed the question of interest [25].
- Moderate: More than one non-critical weakness: the systematic review had more than one weakness but no critical flaws. It may provide an accurate summary of the results of the available studies that were included in the review [25].
- Low: One critical flaw with or without non-critical weaknesses: the review had a critical flaw and may not provide an accurate and comprehensive summary of the available studies that addressed the question of interest [25].
- Critically low: More than one critical flaw with or without non-critical weaknesses: the review had more than one critical flaw and should not be relied on to provide an accurate and comprehensive summary of the available studies [25].

**Table 1:** AMSTAR2 classification of the included studies

Study	Q1	Q2*	Q3	Q4*	Q5	Q6	Q7*	Q8	Q9*	Q10	Q11*	Q12	Q13*	Q14	Q15*	Q16	AMSTAR-2 overall quality
ALESSANDRO RIZZO, 2020 [22]	Y	N	N	Y	N	Y	N	PY	PY	N	Y	Y	Y	Y	N	Y	Critically low
Lawrence Chen, 2016 [16]	Y	Y	Y	Y	N	Y	N	PY	PY	N	N	N	Y	Y	N	Y	Critically low
Ting Zheng, 2020 [19]	Y	N	N	PY	N	Y	N	PY	PY	N	N	Y	Y	Y	Y	Y	Critically low
Xin ZHUANG, 2017 [21]	Y	N	N	PY	N	Y	Y	PY	PY	N	Y	Y	Y	Y	Y	Y	Low
Heng Liu, 2014 [18]	Y	N	Y	PY	N	Y	Y	PY	PY	N	Y	Y	Y	Y	Y	Y	Low
Sheng Zhao, 2016 [17]	Y	N	N	Y	N	Y	N	PY	PY	N	Y	Y	Y	Y	Y	Y	Critically low
Alessandro Rizzo, 2022 [26]	Y	N	N	Y	Y	Y	N	PY	PY	N	N	Y	Y	Y	N	Y	Critically low
Wen-Jie Ma,2020 [27]	Y	N	Y	Y	Y	Y	N	Y	PY	N	Y	Y	Y	Y	Y	Y	Critically low
Julien Edeline, 2022 [28]	Y	N	Y	N	N	N	N	Y	N	N	N	Y	Y	N	N	Y	Critically low
Abdel-Rahman O, 2018 [29]	Y	Y	Y	Y	Y	Y	Y	Y	Y	Y	Y	Y	Y	Y	Y	Y	High
Yan Li, 2019 [30]	Y	N	N	Y	Y	Y	N	Y	N	N	N	N	N	N	Y	Y	Critically low
Jie Ying, 2019 [31]	Y	PY	N	Y	N	Y	N	PY	PY	N	N	Y	N	N	Y	Y	Critically low
Wei Zheng, 2019 [32]	Y	N	N	Y	N	Y	N	PY	N	N	N	N	N	Y	Y	Y	Critically low
Yanfeng Jiang, 2021 [33]	Y	N	Y	PY	N	Y	N	PY	PY	N	N	N	Y	Y	Y	Y	Critically low

AMSTAR-2 items: Q1: Did the research questions and inclusion criteria for the review include the components of PICO? Q2: Did the report of the review contain an explicit statement that the review methods were established prior to the conduct of the review, and did the report justify any signifificant deviations from the protocol? Q3: Did the review authors explain their selection of the study designs for inclusion in the review? Q4: Did the review authors use a comprehensive Literature search strategy? Q5: Did the review authors perform study selection in duplicate? Q6: Did the review authors perform data extraction in duplicate? Q7: Did the review authors provide a list of excluded studies and justify the exclusions? Q8: Did the review authors describe the included studies in adequate detail? Q9: Did the review authors use a satisfactory technique for assessing the risk of bias (RoB) in individual studies that were included in the review? Q10: Did the review authors report on the sources of funding for the studies included in the review? Q11: If meta-analysis was performed, did the review authors use appropriate methods for statistical combination of results? Q12: If meta-analysis was performed, did the review authors assess the potential impact of RoB in individual studies on the results of the meta-analysis or other evidence synthesis? Q13: Did the review authors account for RoB in primary studies when interpreting/discussing the results of the review? Q14: Did the review authors provide a satisfactory explanation for, and discussion of, any heterogeneity observed in the results of the review? Q15: If they performed quantitative synthesis, did the review authors carry out an adequate investigation of publication bias (small study bias) and discuss its likely impact on the results of the review? Q16: Did the review authors report any potential sources of conflflict of interest, including any funding they received for conducting the review?

#### Quality evaluation of evidence

We used the online tool GRADEpro GDT to evaluate the quality of the evidence. GRADE is a grading method for level of evidence and strength of recommendation introduced by the GRADE Working Group in 2004. It can also be used to classify the evidence for intervention SRoMAs [34]. This grading tool classifies the quality of evidence into four grades (High, Moderate, Low, and Very low) according to the type of studies, five degrading factors (risk of bias, indirectness, inconsistency, imprecision, and publication bias), and three upgrading factors (large effect, dose–response gradient, and plausible confounding).

The detailed assessment criteria of GRADE for systematic review and meta-analysis are as follows:

- Study type: Evidence based on randomized controlled studies was initially defined as high level; evidence based on retrospective studies was initially defined as low level.
- Risk of bias: If relevant evidence was from studies with high risk of bias, the quality level of randomized trials and observational studies may be reduced. No serious limitations – Rating down 0 level; Severe limitations – Rating down 1 level; extremely severe limitations – Rating down 2 levels [35].
- Indirectness: There was a large difference or no direct comparison between the populations, interventions, or outcomes in relevant systematic review.– Rating down 1 level [36].
- Inconsistency: After discussing the prior hypothesis that may explain the source of heterogeneity, the research results remained inconsistent (heterogeneity).– Rating down 1 level [37];
- Imprecision: Outcomes conformed to the OIS standard and the corresponding 95% CIs did not contain invalid values.– Precision – Rating down 0 level; Outcomes did not conform to the OIS standard.– Imprecision – Rating down 1 level; Outcomes conformed to the OIS standard, but the corresponding 95% CI contained invalid value; the CI did not exclude significant benefits or hazards. – Imprecision – Rating down 1 level [38].
- Publication bias: If suspected, the quality of evidence should be rated down at least 1 level [39].
- Large effect: The relative risk of direct evidence was large (RR =  –5 or RR = 0.5–0.2) without reasonable confounding. – Large effect – Rating up 1 level; The relative risk of direct evidence was very large (RR > 5 or RR < 0.2) and had no risk of bias or serious problems related to accuracy.– Very large effect – Rating up 2 levels [40];
- Dose–response gradient: Rating up 1 level [40];
- Plausible confounding: Reasonable residual confounding will further support the conclusion of efficacy inference. – Rating up 1 level [40];

#### Data synthesis

We did not conduct re-analyses for this study. We only extracted data from the included studies. The specific data extraction items have been listed above.

Excessive significance test was used to evaluate whether the conclusions of each study have excessive statistical significance [41]. In other words, the number of studies actually observed with significant results (O) (*P* < 0.05) was statistically compared with the number of expected significant results (E). However, most SRoMAs did not test the excessive significance of the conclusions. Our study used the R language “Metaumbrella” package to calculate the “O” and “E” values of each research result. When the calculated “O” value was greater than the “E” value and the corresponding *P*-value was < 0.10, this result was considered to have excessive statistical significance.

Overall, we have presented and discussed important limitations within the evidence base and considered the possible influence of publication biases and excessive statistical significance on review findings.

## Results

### Description of included reviews

**Fig.1:**
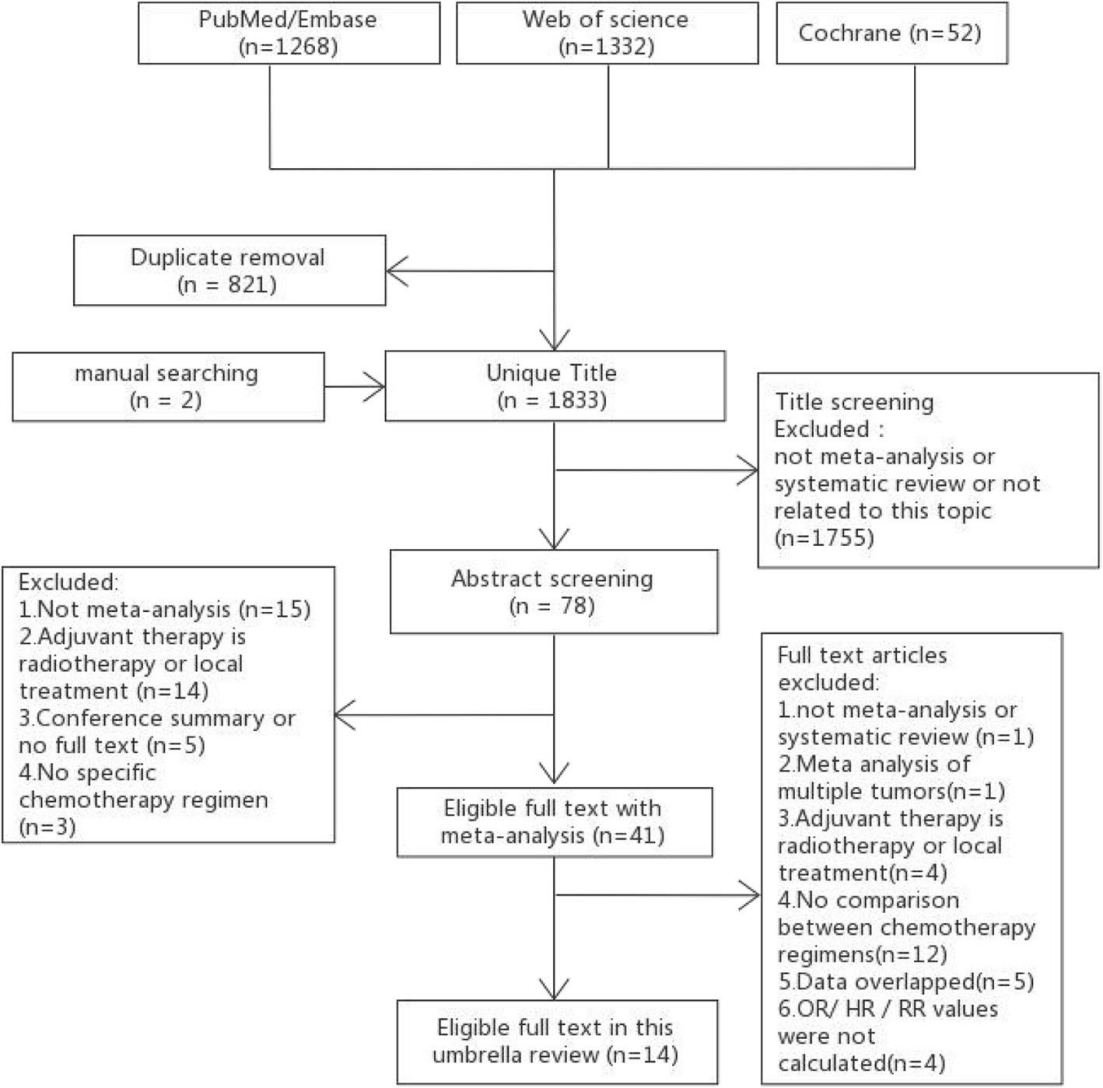
Flow chart of literature screening in this study

The process of literature screening is shown in Fig. 1. Two authors searched 2652 studies independently and systematically. Overall, 1833 articles were included in the initial review after 821 duplicates were removed. After abstract screening and full-text screening, 14 reviews were included in this study. Table S3 lists all the references that were excluded after full-text screening and the corresponding reasons for exclusion. Table 2 shows the basic characteristics of the 14 reviews.

**Table 2:** The general characteristics of the 14 systematic reviews and meta-analyses

First author, Year	Original article retrieval time	Journal	Total No. of included studies	Type of study	Study design	Type of Chemotherapy	Subgroup No. of included studies	Intervention (No.of cases)	Control (No.of cases)	Sample size
ALESSANDRO RIZZO,2020 [22]	2019/11/02	IN VIVO	4	Systematic Review and Meta-analysis	RCTs	G-based + anti-EGFR vs. G-based	4	Gem-based + anti-EGFR (228)	Gem-based CHT alone (222)	450 patients
Lawrence Chen, 2016 [16]	2016/04	MEDICINE	15	Meta-analysis	RCTs	GP vs. G	2	GP(202)	G(184)	386 patients
						GP + anti-EGFR vs. GP	4	GP + anti-EGFR (316)	GP(313)	629 patients
Ting Zheng, 2020 [19]	2018/12	ONCOLOGY RESEARCH AND TREATMENT	5	Meta-analysis	Restrospective; RCTs	GP vs. FP	5	GP(311)	FP(416)	727 patients
Xin ZHUANG, 2017 [21]	2016/07	JOURNAL OF HUAZHONG UNIVERSITY OF SCIENCE ANDTECHNOLOGY-MEDICAL SCIENCES	7	Meta-analysis	RCTs	G-based + anti-EGFR vs. G-based	6	Gem-based + anti-EGFR (432)	Gem-based CHT alone (423)	855 patients
Heng Liu, 2014 [18]	2013/11	WORLD JOURNAL OF GASTROENTEROLOGY	7	Meta-analysis	RCTs	G-based vs. non-G-based	4	Gem-based(150)	non-G-based(153)	303 patients
						G-based vs. G	3	Gem-based(275)	G (280)	555 patients
Sheng Zhao, 2016 [17]	2016/03	ONCOTARGETS AND THERAPY	6	Systematic Review and Meta-analysis	RCTs	G-based + anti-VEGFR/EGFR vs. G-based	6	G-based + anti-VEGFR/ EGFR(NA)	G-based(NA)	855 patients
Alessandro Rizzo, 2022 [26]	2021/12/08	EXPERT REVIEW OF GASTROENTEROLOGY & HEPATOLOGY	2	Meta-analysis	RCTs	Fluoropyrimidine-based doublet CHT vs. ASC or 5-FU/LV	2	Fluoropyrimidine-based doublet CHT(169)	ASC or 5-FU/LV(167)	336 patients
Wen-Jie Ma, 2020 [27]	2019/06/12	HBP	5	Meta-analysis	RCTs	Fluoropyrimidine-based vs. Observation	4	Fluoropyrimidine-based (381)	Observation(358)	739 patients
						G-based vs. Observation	3	G-based(246)	Observation(238)	484 patients
Julien Edeline, 2022 [28]	NA	EUROPEAN JOURNAL OF CANCER	2	Meta-analysis	RCTs	G-based vs. Observation	2	G-based(212)	Observation(207)	419 patients
Abdel-Rahman O, 2018 [29]	2017/06	COCHRANE DATABASE OF SYSTEMATIC REVIEWS	7	Meta-analysis	RCTs	G + S-1 vs. S-1	2	G + S-1(76)	S-1(75)	151 patients
Yan Li,2019 [30]	2018/10/06	FRONTIERS IN ONCOLOGY	25	Network Meta-analysis	Restrospective; RCTs	Folfox-4 vs. Observation	NA	Folfox-4(NA)	Observation(NA)	NA
						XP vs. GP	NA	XP(NA)	GP(NA)	NA
						G + S-1 vs. GC	NA	GS(NA)	GC(NA)	NA
Jie Ying, 2019 [31]	2018/04	CRITICAL REVIEWS IN ONCOLOGY HEMATOLOGY	32	Network Meta-analysis	Restrospective; RCTs	G-based CHT vs. single CHT(mainly FU alone)	15	GEM-based CHT(233)	single CHT(396)	629 patients
						Fluoropyrimidine-based CHT vs. single TAs	15	Fluoropyrimidine-based CHT(527)	single TAs(150)	677 patients
						Taxanes-based CHT vs. single CHT(mainly FU alone)	9	Taxanes-based CHT(54)	single CHT(396)	450 patients
						Fluoropyrimidine-based CHT vs. single CHT(mainly FU alone)	8	Fluoropyrimidine-based CHT (244)	single CHT(348)	592 patients
Wei Zheng, 2019 [32]	2017/11	JOURNAL OF CANCER	16	Network Meta-analysis	RCTs	G + S-1 vs. G	NA	G + S-1(NA)	G(NA)	NA
						G + S-1 vs. 5-FU	NA	G + S-1(NA)	5-FU(NA)	NA
						CapC vs.5-FU	NA	CapC(NA)	5-FU(NA)	NA
						GEMOX vs. 5-FU	NA	GEMOX (NA)	5-FU(NA)	NA
						FP vs. 5-FU	NA	FP(NA)	5-FU(NA)	NA
Yanfeng Jiang, 2021 [33]	2020/08/10	FRONTIERS IN ONCOLOGY	24	Network Meta-analysis	RCTs	Observation vs. Folfox-4	NA	Observation(NA)	Folfox-4(NA)	NA
						Observation vs. C-GEMOX	NA	Observation(NA)	C-GEMOX(NA)	NA
						Observation vs. GEMOX + erlotinib	NA	Observation(NA)	GEMOX + erlotinib(NA)	NA
						GP + cediranib vs. 5-FU	NA	GP + cediranib(NA)	5-FU(NA)	NA
						GP vs. 5-FU	NA	GP(NA)	5-FU(NA)	NA
						G + S-1 vs. 5-FU	NA	GS(NA)	5-FU(NA)	NA
						C-GEMOX vs. 5-FU	NA	C-GEMOX(NA)	5-FU(NA)	NA
						RAM + GP vs. 5-FU	NA	RAM + GP(NA)	5-FU(NA)	NA
						MER + GP vs. 5-FU	NA	MER + GP(NA)	5-FU(NA)	NA
						XELOX vs. G + XELOX	NA	XELOX(NA)	G + XELOX(NA)	NA
						XELOX vs. GEMOX + erlotinib	NA	XELOX(NA)	GEMOX + erlotinib(NA)	NA

### Objectives and scope of the reviews

All included reviews aimed to evaluate the safety and effectiveness of various chemotherapy schemes for the treatment of advanced biliary tract cancer.

Among 14 included reviews:

- Four reviews investigated the efficacy and safety of gemcitabine-based chemotherapy + targeted therapy versus gemcitabine-based chemotherapy [16, 17, 21, 22];
- Two reviews reported the comparison between gemcitabine-based combination chemotherapy and gemcitabine monotherapy [16, 18];
- One review reported the comparison between fluoropyrimidine + cisplatin and gemcitabine + cisplatin [19];
- Two reviews reported the comparisons between gemcitabine-containing and gemcitabine-free chemotherapy [18, 29];
- Two reviews examined the role of fluorouracil-based second-line chemotherapy in biliary tract cancer [26, 27];
- Two reviews reported the comparison between gemcitabine-based chemotherapy and observation [25, 28];
- Four reviews reported the comparison between chemotherapy and targeted therapy regimens in the network meta-analysis [30–33].

### Study characteristics and populations

All SRoMAs were published between 2014 and 2022. Of the 14 reviews, 11 contained only randomized controlled studies, and 3 included both randomized controlled studies and cohort studies. The number of original studies included in these reviews ranged from 2 to 32. These reviews included the efficacy and/or safety outcomes of 36 chemotherapy or targeted therapy regimens. The number of cases included in these studies ranged from 151 to 855. However, the exact numbers of cases were unavailable in some studies.

### Methodological quality of included systematic reviews

We used the AMSTAR2 rating scale to assess the methodological quality in each included review. The results of these reviews were classified into four grades (High, Moderate, Low, and Critically low). Most of the studies did not register in advance and did not provide a literature exclusion list, which failed to meet the key items 2 and 7 of the AMSTAR2 scale. Thus, their methodological quality was directly rated as critically low.

Overall, the vast majority (11 studies, 78.6%) of methodological qualities of the reviews were assessed as critically low (Tables 1 and 3). Two reviews were assessed as low, and one was assessed as high.

**Table 3:** AMSTAR2 and GRADE classification of the evidence

First author, Year	Type of Chemotherapy	Clinical Outcome	MA metric	AMSTAR2	GRADE
ALESSANDRO RIZZO,2020 [22]	G-based + anti-EGFR vs. G-based	OS	HR	Critically low	Low
		PFS	HR	Critically low	Low
		ORR	RR	Critically low	Very Low
		Toxicities–Neutropenia	RR	Critically low	Low
		Toxicities–Thrombocytopenia	RR	Critically low	Very Low
		Toxicities–Skin rash	RR	Critically low	Moderate
		Toxicities–Diarrhea	RR	Critically low	Very Low
		Toxicities–Fatigue	RR	Critically low	Very Low
Lawrence Chen,2016 [16]	GP vs. G	Duration of OS	MD	Critically low	Low
		OS	HR	Critically low	Low
		Duration of PFS	MD	Critically low	Low
		PFS	HR	Critically low	Low
		ORR	OR	Critically low	Low
	GP + anti-EGFR vs. GP	Duration of OS	MD	Critically low	Low
		OS	HR	Critically low	Low
		Duration of PFS	MD	Critically low	Low
		PFS	HR	Critically low	Low
		ORR	OR	Critically low	Low
Ting Zheng,2020 [19]	GP vs. FP	ORR	RR	Critically low	Very Low
		DCR	RR	Critically low	Very Low
		PFS/TTP	HR	Critically low	Low
		OS	HR	Critically low	Low
		Toxicities–Neutropenia	NA	Critically low	Very Low
		Toxicities–Anemia	NA	Critically low	Very Low
		Toxicities–Trombocytopenia	NA	Critically low	Very Low
		Toxicities–Nausea/Vomiting	NA	Critically low	Very Low
		Toxicities–Anorexia	NA	Critically low	Very Low
		Toxicities–Nephropathy	NA	Critically low	Very Low
		Toxicities–Neuropathy	NA	Critically low	Very Low
Xin ZHUANG,2017 [21]	G-based + anti-EGFR vs. G-based	Toxicities–Neutropenia	OR	Low	Very Low
		Toxicities–Thrombocytopenia	OR	Low	Very Low
		Toxicities–Anemia	OR	Low	Very Low
		Toxicities–Peripheral neuropathy	OR	Low	Very Low
		Toxicities–Increased AST/ALT	OR	Low	Very Low
Heng Liu,2014 [18]	G-based vs. non-G-based	DRR	OR	Low	Low
		DCR	OR	Low	Very Low
		PFS	OR	Low	Low
		OS	OR	Low	Low
		Toxicities–Leukopenia	OR	Low	Moderate
		Toxicities–Anemia	OR	Low	High
		Toxicities–Neutropenia	OR	Low	Very Low
		Toxicities–Thrombocytopenia	OR	Low	Very Low
		Toxicities– Increased ALT level	OR	Low	Very Low
	G-based vs. G	Toxicities–Leukopenia	OR	Low	Low
		Toxicities–Anemia	OR	Low	Very Low
		Toxicities–Neutropenia	OR	Low	Low
		Toxicities–Thrombocytopenia	OR	Low	Very Low
		Toxicities– Increased ALT level	OR	Low	Very Low
Sheng Zhao,2016 [17]	G-based + anti-VEGFR/EGFR vs. G-based	Toxicities– Nausea	RR	Critically low	Very Low
		Toxicities– Vomiting	RR	Critically low	Very Low
		Toxicities– Diarrhea	RR	Critically low	Moderate
Alessandro Rizzo,2022 [26]	Fluoropyrimidine-based doublet CHT vs. ASC or 5-FU/LV	OS	HR	Critically low	Low
		DCR	OR	Critically low	Moderate
		ORR	OR	Critically low	Moderate
Wen-Jie Ma,2020 [27]	Fluoropyrimidine-based vs. Observation	OS	HR	Critically low	Moderate
	G-based vs. Observation	OS	HR	Critically low	Moderate
Julien Edeline,2022 [28]	G-based vs. Observation	RFS-All Patients	HR	Critically low	Low
		RFS-R1 resection Patients	HR	Critically low	Low
		RFS-N + tumor Patients	HR	Critically low	Low
		OS-All Patients	HR	Critically low	Low
		OS-R1 resection Patients	HR	Critically low	Low
		OS-N + tumor Patients	HR	Critically low	Low
Abdel-Rahman O,2018 [29]	G + S-1 vs. S-1	All-cause mortality at 1 year	RR	High	Very Low
		ORR(S-1 vs.G + S-1)	RR	High	Moderate
		Toxicities–Grade 1—4 Anaemia	RR	High	Very Low
		Toxicities–Grade 1—4 Thrombocytopenia	RR	High	Moderate
		Toxicities–Grade 1—4 Neutropenia	RR	High	Moderate
		Toxicities–Febrile Neutropenia	RR	High	Very Low
Yan Li,2019 [30]	FOLFOX-4 vs. Observation	OS	HR	Critically low	—
	XP vs. GP	OS	HR	Critically low	—
	G + S-1 vs. GC	OS	HR	Critically low	—
Jie Ying,2019 [31]	G-based CHT vs. single CHT(mainly FU alone)	DCR	RR	Critically low	—
	Fluoropyrimidine-based CHT vs. single TAs	DCR	RR	Critically low	—
	Taxanes-based CHT vs. single CHT(mainly FU alone)	DCR	RR	Critically low	—
	Fluoropyrimidine-based CHT vs. single CHT(mainly FU alone)	1-year OS	RR	Critically low	—
Wei Zheng,2019 [32]	G + S-1 vs. G	ORR	OR	Critically low	—
		OS	HR	Critically low	—
	G + S-1 vs. 5-FU	ORR	OR	Critically low	—
		OS	HR	Critically low	—
	CapC vs.5-FU	ORR	OR	Critically low	—
	GEMOX vs. 5-FU	OS	HR	Critically low	—
	FP vs. 5-FU	OS	HR	Critically low	—
Yanfeng Jiang,2021 [33]	Observation vs. Folfox-4	PFS	HR	Critically low	—
	Observation vs. C-GEMOX	PFS	HR	Critically low	—
	Observation vs. GEMOX + erlotinib	PFS	HR	Critically low	—
	GP + cediranib vs. 5-FU	ORR (5-FU vs.GP + cediranib)	OR	Critically low	—
		Toxicities–Neutropenia	OR	Critically low	—
	GP vs. 5-FU	Toxicities–Neutropenia	OR	Critically low	—
	G + S-1 vs. 5-FU	Toxicities–Neutropenia	OR	Critically low	—
	C-GEMOX vs. 5-FU	Toxicities–Neutropenia	OR	Critically low	—
	RAM + GP vs. 5-FU	Toxicities–Neutropenia	OR	Critically low	—
	MER + GP vs. 5-FU	Toxicities–Neutropenia	OR	Critically low	—
	XELOX vs. G + XELOX	Toxicities–Vomiting	OR	Critically low	—
	XELOX vs. GEMOX + erlotinib	Toxicities– Diarrhea	OR	Critically low	—

### Certainty of evidence

Among all the evidence we summarized (Table 4), 36 items (38.3%) presented low heterogeneity (I^2^ < 25%); 13 showed moderate heterogeneity (25% < I^2^ < 75%); 9 showed high heterogeneity (I^2^ > 75%); and 3 only reported no significant heterogeneity. Furthermore, 32 items did not report heterogeneity. For evidence with significant heterogeneity (*p* < 0.05), the quality of evidence will be rated down 1 level.

**Table 4:** Comparison of efficacy and safety of different chemotherapy or targeted therapy regimens in patients with biliary tract cancer

First author, Year	Type of Chemotherapy	Study design	Clinical Outcome	Effects model	MA metric	Estimates	95%CI-low	95%CI-up	*p*-value	*I*^*2*^%	*P*-value for TES	*P*-value forEgger test	MA Quality Assessment
ALESSANDRO RIZZO,2020 [22]	G-based + anti-EGFR vs. G-based	RCTs	OS	Random	HR	0.82	0.64	1.06	0.13	33	0.2472	NA	Cochrane ROB Tool
			PFS	Fixed	HR	0.88	0.73	1.08	0.22	0	1	NA	
			ORR	Fixed	RR	1.34	0.91	1.99	0.14	0	1	NA	
			Toxicities–Neutropenia	Fixed	RR	1.95	1.13	3.36	0.02	0	0.2367	NA	
			Toxicities–Thrombocytopenia	Fixed	RR	1.69	0.99	2.87	0.05	0	1	NA	
			Toxicities–Skin rash	Fixed	RR	18.11	5.13	63.91	< 0.00001	0	0.8343	NA	
			Toxicities–Diarrhea	Fixed	RR	1.65	0.89	3.04	0.11	0	1	NA	
			Toxicities–Fatigue	Fixed	RR	2.01	0.91	4.44	0.09	0	1	NA	
Lawrence Chen,2016 [16]	GP vs. G	RCTs	Duration of OS	Random	WMD	-3.52	-5.14	-1.35	0.0008	0	—	NA	Cochrane ROB Tool
			OS	Random	HR	0.65	0.53	0.79	< 0.00001	0	0.8264	NA	
			Duration of PFS	Random	WMD	-2.60	-3.81	-1.40	< 0.00001	0	—	NA	
			PFS	Random	HR	0.63	0.52	0.76	< 0.00001	0	0.8544	NA	
			ORR	Random	OR	0.53	0.31	0.88	0.02	0	0.6220	NA	
	GP + anti-EGFR vs. GP		Duration of OS	Random	WMD	-1.49	-2.56	-0.43	0.006	0	—	NA	
			OS	Random	HR	0.90	0.70	1.15	0.39	0	1	NA	
			Duration of PFS	Random	WMD	-0.07	-1.91	1.77	0.94	0	—	NA	
			PFS	Random	HR	0.79	0.63	0.99	0.04	0	1	NA	
			ORR	Random	OR	0.56	0.38	0.83	0.003	0	0.9783	NA	
Ting Zheng,2020 [19]	GP vs. FP	Restrospective;RCTs	ORR	Fixed	RR	1.13	0.80	1.58	> 0.05	44.9	0.482	0.187	Cochrane ROB Tool; NOS
			DCR	Fixed	RR	1.02	0.91	1.13	> 0.05	12	0.7508	0.209	
			PFS/TTP	Fixed	HR	0.95	0.86	1.05	> 0.05	0	1	NA	
			OS	Fixed	HR	1.06	0.98	1.14	> 0.05	37.3	0.2265	NA	
			Toxicities–Neutropenia	NA	NA	16.7 (9.3–25.8) vs. 19.3 (3.7–43.3)			< 0.001	91.4	—	NA	
			Toxicities–Anemia	NA	NA	5.6 (1.1–13.3) vs. 13.1 (7.8–19.5)			< 0.001	81.5	—	NA	
			Toxicities–Trombocytopenia	NA	NA	6 (2.7–10.5) vs. 10.3 (2.7–22.1)			< 0.001	79.5	—	NA	
			Toxicities–Nausea/Vomiting	NA	NA	5.7 (4–7.7) vs. 7.8 (5.6–10.3)			< 0.001	0	—	NA	
			Toxicities–Anorexia	NA	NA	2.2 (1–3.7) vs. 3.1 (0.2–9.3)			< 0.001	64.9	—	NA	
			Toxicities–Nephropathy	NA	NA	1.1 (0.2–2.7) vs. 2.9 (0.7–6.6)			< 0.001	67.6	—	NA	
			Toxicities–Neuropathy	NA	NA	0.9 (0.3–1.8) vs. 2.6 (1.4–4.1)			< 0.001	47.9	—	NA	
Xin ZHUANG,2017 [21]	G-based + anti-EGFR vs. G-based	RCTs	Toxicities–Neutropenia	Fixed	OR	1.37	0.89	2.12	0.15	0	0.3165	NA	Jadad Scale
			Toxicities–Thrombocytopenia	Fixed	OR	1.4	0.83	2.39	0.21	48	1	NA	
			Toxicities–Anemia	Fixed	OR	1.21	0.62	2.38	0.57	0	1	NA	
			Toxicities–Peripheral neuropathy	Fixed	OR	1.52	0.81	2.88	0.19	0	0.2096	NA	
			Toxicities–Increased AST/ALT	Fixed	OR	1.4	0.82	2.39	0.22	0	1	NA	
Heng Liu,2014 [18]	G-based vs. non-G-based	RCTs	DRR	Fixed	OR	1.39	0.81	2.40	0.24	48	0.8733	1.00	Jadad Scale
			DCR	Random	OR	1.48	0.43	5.07	0.53	81	0.8312	0.23	
			PFS(months)	Random	OR	1.78	-0.39	3.96	0.11	99	—	0.55	
			OS(months)	Random	OR	1.51	-1.37	4.38	0.3	99	—	1.00	
			Toxicities–Leukopenia	Random	OR	7.17	1.43	36.08	0.02	64	0.8917	NA	
			Toxicities–Anemia	Fixed	OR	7.04	2.59	19.12	0.00001	0	0.1356	NA	
			Toxicities–Neutropenia	Random	OR	4.63	0.95	22.50	0.06	82	0.9924	NA	
			Toxicities–Thrombocytopenia	Random	OR	2.79	0.66	11.81	0.16	70	0.3348	NA	
			Toxicities– Increased ALT level	Fixed	OR	1.11	0.56	2.23	0.76	46	1	NA	
	G-based vs. G		Toxicities–Leukopenia	Random	OR	1.82	1.13	2.94	0.01	0	1	NA	
			Toxicities–Anemia	Fixed	OR	1.96	1.07	3.62	0.03	54	0.3617	NA	
			Toxicities–Neutropenia	Random	OR	1.78	1.19	2.66	0.005	0	0.6637	NA	
			Toxicities–Thrombocytopenia	Random	OR	1.13	0.6	2.14	0.71	0	1	NA	
			Toxicities– Increased ALT level	Fixed	OR	0.76	0.47	1.25	0.29	66	1	NA	
Sheng Zhao,2016 [17]	G-based + anti-VEGFR/EGFR vs. G-based	RCTs	Toxicities– Nausea	NA	RR	1.01	0.41	2.47	0.98	No significant	—	NA	Jadad Scale
			Toxicities– Vomiting	NA	RR	0.71	0.31	1.60	0.41	No significant	—	NA	
			Toxicities– Diarrhea	NA	RR	2.48	1.2	5.10	0.014	No significant	—	NA	
Alessandro Rizzo,2022 [26]	Fluoropyrimidine-based doublet CHT vs. ASC or 5-FU/LV	RCTs	OS	Fixed	HR	0.63	0.49	0.80	< 0.0001	0	0.5867	NA	Cochrane ROB Tool
			DCR	Fixed	OR	5.18	3.3	10.23	NA	84	0.9772	NA	
			ORR	Random	OR	3.24	1.18	8.92	NA	0	1	NA	
Wen-Jie Ma,2020 [27]	Fluoropyrimidine-based vs. Observation	RCTs	OS	Random	HR	0.83	0.7	0.99	0.04	13	0.5350	0.62	Jadad Scale
	G-based vs. Observation		OS	Random	HR	0.91	0.74	1.12	0.37	2	1	0.62	
Julien Edeline,2022 [28]	G-based vs. Observation	RCTs	RFS-All Patients	NA	HR	0.91	0.71	1.16	0.46	NA	—	NA	NA
			RFS-R1 resection Patients	NA	HR	1.10	0.58	2.07	0.77	NA	—	NA	
			RFS-N + tumor Patients	NA	HR	0.86	0.60	1.23	0.40	NA	—	NA	
			OS-All Patients	NA	HR	1.03	0.78	1.35	0.85	NA	—	NA	
			OS-R1 resection Patients	NA	HR	1.25	0.63	2.49	0.52	NA	—	NA	
			OS-N + tumor Patients	NA	HR	0.99	0.67	1.46	0.94	NA	—	NA	
Abdel-Rahman O,2018 [29]	G + S-1 vs. S-1	RCTs	All-cause mortality at 1 year	Random	RR	0.61	0.33	1.13	0.12	76	0.2928	NA	Cochrane ROB Tool
			ORR(S-1 vs.G + S-1)	Random	RR	2.46	1.27	4.57	0.0073	0	0.5223	NA	
			Toxicities–Grade 1—4 Anaemia	Random	RR	1.26	1.00	1.59	0.052	0	1	NA	
			Toxicities–Grade 1—4 Thrombocytopenia	Random	RR	2.45	1.39	4.32	0.0019	0	0.6803	NA	
			Toxicities–Grade 1—4 Neutropenia	Random	RR	3.30	1.04	10.50	0.043	66	0.4714	NA	
			Toxicities–Febrile Neutropenia	Random	RR	2.97	0.32	27.87	0.34	0	1	NA	
Yan Li,2019 [30]	Observation vs. FOLFOX-4	Restrospective;RCTs	OS	NA	HR	3.40	1.70	6.70	NA	NA	—	NA	NA
	XP vs. GP		OS	NA	HR	0.74	0.51	1.10	NA	NA	—	NA	
	G + S-1 vs. GC		OS	NA	HR	1.10	0.71	1.50	NA	NA	—	NA	
Jie Ying,2019 [31]	G-based CHT vs. single CHT(mainly FU alone)	Restrospective;RCTs	DCR	NA	RR	1.36	1.04	1.80	0.012	NA	—	Funnel plot only	NOS
	Fluoropyrimidine-based CHT vs. single TAs		DCR	NA	RR	0.78	0.61	1.00	0.03	NA	—	Funnel plot only	
	Taxanes-based CHT vs. single CHT(mainly FU alone)		DCR	NA	RR	1.54	1.02	2.32	0.02	NA	—	Funnel plot only	
	Fluoropyrimidine-based CHT vs. single CHT(mainly FU alone)		1-year OS	NA	RR	0.51	0.29	0.87	0.006	NA	—	Funnel plot only	
Wei Zheng,2019 [32]	G + S-1 vs. G	RCTs	ORR	NA	OR	4.72	1.31	17.02	NA	NA	—	Funnel plot only	NA
			OS	NA	HR	0.43	0.20	0.93	NA	NA	—	Funnel plot only	
	G + S-1 vs. 5-FU		ORR	NA	OR	9.08	1.56	89.20	NA	NA	—	Funnel plot only	
			OS	NA	HR	0.51	0.28	0.96	NA	NA	—	Funnel plot only	
	CapC vs.5-FU		ORR	NA	OR	5.46	1.07	56.63	NA	NA	—	Funnel plot only	
	GEMOX vs. 5-FU		OS	NA	HR	0.57	0.32	0.96	NA	NA	—	Funnel plot only	
	FP vs. 5-FU		OS	NA	HR	1.88	1.07	3.16	NA	NA	—	Funnel plot only	
Yanfeng Jiang,2021 [33]	Observation vs. Folfox-4	Restrospective;RCTs	PFS	NA	HR	2.88	1.05	7.93	NA	NA	—	Funnel plot only	Cochrane ROB Tool
	Observation vs. C-GEMOX		PFS	NA	HR	2.82	1.20	6.62	NA	NA	—	Funnel plot only	
	Observation vs. GEMOX + erlotinib		PFS	NA	HR	3.21	1.38	7.56	NA	NA	—	Funnel plot only	
	GP + cediranib vs. 5-FU		ORR (5-FU vs.GP + cediranib)	NA	OR	0.13	0.02	0.87	NA	NA	—	Funnel plot only	
			Toxicities–Neutropenia	NA	OR	0.04	0	0.65	NA	NA	—	Funnel plot only	
	GP vs. 5-FU		Toxicities–Neutropenia	NA	OR	0.06	0.01	0.50	NA	NA	—	Funnel plot only	
	G + S-1 vs. 5-FU		Toxicities–Neutropenia	NA	OR	0.05	0	0.55	NA	NA	—	Funnel plot only	
	C-GEMOX vs. 5-FU		Toxicities–Neutropenia	NA	OR	0.08	0.01	0.60	NA	NA	—	Funnel plot only	
	RAM + GP vs. 5-FU		Toxicities–Neutropenia	NA	OR	0.03	0	0.38	NA	NA	—	Funnel plot only	
	MER + GP vs. 5-FU		Toxicities–Neutropenia	NA	OR	0.03	0	0.41	NA	NA	—	Funnel plot only	
	XELOX vs. G + XELOX		Toxicities–Vomiting	NA	OR	0.07	0	0.98	NA	NA	—	Funnel plot only	
	XELOX vs. GEMOX + erlotinib		Toxicities– Diarrhea	NA	OR	0.09	0.01	0.63	NA	NA	—	Funnel plot only	

Egger’s test was used to summarize publication bias or small study effects in SRoMAs. Of the 14 SRoMAs, 8 studies did not measure publication bias, and the remaining did not report significant publication bias (Table 4).

Table 4 illustrates the results of the test for excess significance for each outcome. Overall, 17 out of 94 outcomes had a greater number of observed positive studies than the number of expected positive studies. None of the outcomes had statistical evidence (*p* < 0.1) of excess statistical significance.

After assessing the quality of evidence for each outcome, only 1 outcome was rated as “High” quality, 10 were rated as “Moderate” quality, 27 were rated as “Low” quality, and 30 were rated as “Very Low” quality (Tables S4 and Table 3). In addition, because the evidence summary table (NMA-SoF) for continuous variables of the network meta-analysis had not been tested by the GRADE working group, we did not evaluate the quality of evidence for the network meta-analyses in this study.

### Effect of interventions

#### Gemcitabine-based Chemotherapy + Targeted Therapy versus Gemcitabine-based Chemotherapy

##### Primary outcomes

Unknown benefit or harm or no effect or equivalence:

ALESSANDRO RIZZO ]14] found no significant differences in OS (HR = 0.82, 95% CI 0.64–1.06, GRADE: Low), PFS (HR = 0.88, 95% CI 0.73–1.08, GRADE: Low), and ORR (RR = 1.34, 95% CI 0.91–1.99, GRADE: Very Low) between the experimental group (chemotherapy + targeted group) and the control group (chemotherapy group). However, one meta-analysis conducted by Lawrence Chen et al. [16] showed a significant difference in OS (Duration of OS: WMD = -1.49, 95% CI -2.56–0.43, GRADE: Low) between the two groups but no difference in PFS (Duration of PFS:WMD = -0.07, 95% CI -1.91–1.77, GRADE: Low). Chen et al. [16] also reported that the ORR of the experimental group (OR = 0.56, 95% CI 0.38–0.83, GRADE: Low) was significantly better than that of the control group (Tables 3 and 4).

##### Secondary outcomes

Clear evidence of harm:

In terms of safety, the following toxic effects were reported: neutropenia, thrombocytopenia, skin rash, nausea, vomiting, diarrhea, fatigue, anemia, peripheral neuropathy, and AST/ALT elevation. Among them, only the risks of neutropenia [22] (RR = 1.95, 95% CI 1.13–1.36, GRADE: Low), skin rash [22] (RR = 18.11, 95% CI 5.13–63.91, GRADE: Moderate), and diarrhea [17] (RR = 2.48, 95% CI 1.2–5.10, GRADE: Moderate) were higher in the experimental group than in the control group. However, the risk of neutropenia and diarrhea was inconsistent in different meta-analyses. For example, neutropenia reported by Xin ZHUANG [21] (RR = 1.37, 95% CI 0.89–2.21, GRADE: Very Low) and diarrhea reported by ALESSANDRO RIZZO [22] (RR = 1.65, 95% CI 0.89–3.04, GRADE: Very Low) showed no difference in risk between the two groups (Tables 3 and 4). Given that the former had better outcomes than the latter, we believe that patients receiving chemotherapy + targeted therapy have a higher risk of skin rash and diarrhea than their counterparts.

#### Gemcitabine-based Chemotherapy versus Gemcitabine monotherapy

##### Primary outcomes

Unknown benefit or harm or no effect or equivalence:

Lawrence Chen’s study showed [16] that combination chemotherapy was superior to single-drug chemotherapy in terms of OS (Duration of OS:WMD = -3.52, 95% CI -5.14–1.35;OS:HR = 0.65, 95% CI 0.53–0.79, GRADE: Low), PFS (Duration of PFS:WMD = 2.60, 95% CI 3.81–1.40; PFS:HR = 0.63, 95% CI 0.52–0.76, GRADE: Low), and ORR (OR = 0.53, 95% CI 0.31–0.88, GRADE: Low) (Tables 3 and 4).

##### Secondary outcomes

Unknown benefit or harm or no effect or equivalence:

Among the toxic reactions reported [18], the risks of leukopenia (OR = 1.82, 95% CI 1.13–2.94, GRADE: Low), anemia (OR = 1.96, 95% CI 1.07–3.62), and neutropenia (OR = 1.78, 95% CI 1.19–2.66, GRADE: Low) were higher in the combined chemotherapy group than in the single chemotherapy group. The risk of thrombocytopenia (OR = 1.13, 95% CI 0.60–2.14, GRADE: Very Low) and increased ALT levels (OR = 0.76, 95% CI 0.47–1.25, GRADE: Very Low) showed no difference between groups (Tables 3 and 4).

#### Fluoropyrimidine + Cisplatin versus Gemcitabine + Cisplatin

Fluoropyrimidine drugs mainly include capecitabine, 5-FU, and S-1 in the chemotherapy system for biliary carcinoma.

##### Primary outcomes

Unknown benefit or harm or no effect or equivalence:

Zheng et al. Foundthat fluorouracil + cisplatin and gemcitabine + cisplatin showed no difference in efficacy and prognosis [19] (ORR [RR = 1.13, 95% CI = 0.80–1.58, GRADE: Very Low], DCR [RR = 1.02, 95% CI = 0.91–1.13, GRADE: Very Low], PFS [HR = 0.95, 95% CI 0.86–1.05, GRADE: Low), and OS [HR = 1.06, 95% CI 0.98–1.14, GRADE: Low]) (Tables 3 and 4).

##### Secondary outcomes

Unknown benefit or harm or no effect or equivalence:

The toxic reactions reported included neutropenia (16.7 [9.3–25.8] vs. 19.3 [3.7–43.3]) anemia (5.6 [1.1–13.3] vs. 13.1 [7.8–19.5]), thrombocytopenia (6 [2.7–10.5] vs. 10.3 [2.7–22.1]), nausea and vomiting (5.7 [4–7.7] vs. 7.8 [5.6–10.3]), anorexia (2.2 [1–3.7] vs. 3.1 [0.2–9.3]), nephropathy (1.1 [0.2–2.7] vs. 2.9 [0.7–6.6]), and neuropathy (0.9 [0.3–1.8] vs. 2.6 [1.4–4.1]). The incidence of these complications in the fluoropyrimidine + cisplatin group was significantly higher than that in the gemcitabine + cisplatin group (GRADE: Very Low) (Tables 3 and 4).

#### Gemcitabine-based Chemotherapy versus Non-Gemcitabine-based Chemotherapy

##### Primary outcomes

Unknown benefit or harm or no effect or equivalence:

Heng Liu et al. [18] conducted a meta-analysis of four different studies (gemcitabine + mitomycin C vs. capecitabine + mitomycin C, gemcitabine + oxaliplatin vs. fluorouracil, gemcitabine + cisplatin vs. S-1 + cisplatin, and gemcitabine + S-1 vs. S-1). The results showed that OS (months; OR = 1.51, 95% CI -1.37–4.38, GRADE: Low), PFS (months; OR = 1.78, 95% CI -0.39–3.96, GRADE: Low), DCR (OR = 1.48, 95% CI 0.43–5.07, GRADE: Very Low), and DRR (OR = 1.39, 95% CI 0.81–2.40, GRADE: Low) had no statistical difference between the two groups (Tables 3 and 4).

##### Secondary outcomes

Clear evidence of harm:

Heng Liu et al. [18] reported that the difference in the risk of toxic reactions was statistically significant only in leukopenia (OR = 7.17, 95% CI 1.43–36.08, GRADE: Moderate) and anemia (OR = 7.04, 95% CI 2.59–19.12, GRADE: High), suggesting that gemcitabine-based chemotherapy was more toxic than non-gemcitabine-based chemotherapy.

Unknown benefit or harm or no effect or equivalence:

However, there were no statistically significant differences between the two groups in toxicities such as neutropenia (OR = 4.63, 95% CI 0.95–22.50, GRADE: Very Low), thrombocytopenia (OR = 2.79, 95% CI 0.66–11.81, GRADE: Very Low), and increased ALT levels (OR = 1.11, 95% CI 0.56–2.23, GRADE: Very Low) (Tables 3 and 4).

##### Primary outcomes

Clear evidence of benefit:

Abdel-rahman O [29] only reported the comparison between gemcitabine + S-1 and S-1 monotherapy, finding that the ORR of gemcitabine + S-1 regimen was superior to that of S-1 monotherapy (RR [S-1 vs. G + S-1] = 2.46, 95% CI 1.27–4.57, GRADE: Moderate).

Unknown benefit or harm or no effect or equivalence:

All-cause mortality at 1 year was not different between the two groups (RR = 0.61, 95% CI 0.33–1.13, GRADE: Very Low) (Tables 3 and 4).

##### Secondary outcomes

Clear evidence of harm:

As for the safety comparison, especially in the aspects of thrombocytopenia (RR = 2.45, 95% CI 1.39–4.32, GRADE: Moderate) and neutropenia (RR = 3.30, 95% CI 1.04–10.50, GRADE: Moderate), the toxicity of combined chemotherapy (gemcitabine + S-1) was significantly higher than that of single-drug chemotherapy (S-1) (Tables 3 and 4).

#### Fluoropyrimidine-based Chemotherapy

##### Primary outcomes

Clear evidence of benefit:

Two SRoMAs examined the role of fluorouracil-based second-line chemotherapy in biliary tract cancer (Table 4). Wen-jie Ma et al. [27] compared the efficacy differences between patients receiving fluoropyrimidine-based second-line chemotherapy or optimal supportive therapy after biliary tract cancer surgery and found that receiving fluoropyrimidine-based chemotherapy improved patients’ OS (HR = 0.83, 95% CI 0.7–0.99, GRADE: Moderate). Alessandro Rizzo et al. [26] performed a meta-analysis of two recently published clinical trials to evaluate the role of second-line fluoropyrimidine-based chemotherapy in advanced biliary tract cancer. In addition, higher DCR (0R = 5.18, 95% CI 3.3–10.23, GRADE: Moderate) and ORR (OR = 3.24, 95% CI 1.18–8.92, GRADE: Moderate) were observed in BTC patients receiving fluoropyrimidine-based chemotherapy (Tables 3 and 4).

Unknown benefit or harm or no effect or equivalence:

The study found a significant reduction in the risk of death with fluoropyrimidine-based chemotherapy (HR = 0.63, 95% CI 0.49–0.8, GRADE: Low) (Tables 3 and 4).

#### Gemcitabine-based Chemotherapy versus Observation

##### Primary outcomes

Clear evidence of no effect or equivalence:

Two studies [27, 28] reported the efficacy of gemcitabine-based chemotherapy in patients with postoperative biliary tract cancer (Table 4). Surprisingly, gemcitabine-based chemotherapy did not improve postoperative patients’ OS compared with supportive treatment [27] (HR = 0.91, 95% CI 0.74–1.12, GRADE: Moderate). Similarly, Julien Edeline et al. [28] investigated whether gemcitabine improves patients’ RFS (all patients; HR = 0.91, 95% CI 0.71–1.16, GRADE: Low) and OS (all patients; HR = 1.03, 95%CI 0.78–1.35, GRADE: Low). In addition, they performed subgroup analyses based on whether patients underwent R0 resection (RFS [R1 resection patients] HR = 1.10, 95% CI 0.58–2.07, GRADE: Low; OS [R1 resection patients] HR = 1.25, 95% CI 0.63–2.49, GRADE: Low) and lymph node metastasis (RFS [N + tumor patients] HR = 0.86, 95% CI 0.60–1.23, GRADE: Low; OS (N + tumor patients) HR = 0.99, 95% CI 0.67–1.46, GRADE: Low). However, all results showed no difference in efficacy between gemcitabine-based chemotherapy and supportive therapy in patients after biliary tract cancer surgery (Tables 3 and 4).

#### Comparison between chemotherapy and targeted therapy regimens in the network meta-analysis

Network meta-analysis has the advantage of combining direct and indirect evidence and can compensate for the deficiency of traditional meta-analysis. To further summarize various evidence, in addition to the conventional meta-analysis, four network meta-analyses were included in this study [30–33] (Table 4).

##### Primary Outcomes

Yan Li et al. [30] found in the comparison of 18 chemotherapy schemes and best support therapy (BSC) that the curative effect of FOLFOX-4 chemotherapy (HR = 3.4, 95% CI 1.70–6.70) was the most significant among these schemes. This finding suggests that the FOLFOX-4 regimen may have the potential to be the best chemotherapy regimen for patients with advanced BTC. Gemcitabine + platinum (GP) has been used as a standard first-line chemotherapy scheme for advanced biliary cancer. This study found no significant difference in improving OS between GP, XP (HR = 0.74, 95% CI 0.51–1.10), and G + S-1(HR = 1.10, 95% CI 0.71–1.50) in patients with advanced BTC. Thus, XP and G + S-1 may be used as an alternative to first-line chemotherapy for patients with advanced BTC.

Regarding second-line chemotherapy for advanced BTC, Jie Ying et al. [31] summarized the difference in efficacy and safety between combination therapy and monotherapy. Compared with patients receiving 5-FU monotherapy, patients receiving gemcitabine-based (RR = 1.36, 95%CI 1.04–1.80) or taxane-based (RR = 1.54, 95% CI 1.02–2.32) combination chemotherapy had higher DCR. No significant difference in 1-year OS and ORR was found between the combination therapy group and the monotherapy group. However, fluoropyrimidine-based combination chemotherapy regimen reduced 1-year OS (RR = 0.51, 95% CI 0.29–0.87) compared with single-drug chemotherapy. It also reduced DCR (RR = 0.78, 95% CI 0.61–1.00) compared with single-drug targeted therapy.

Wei Zheng et al. [32] conducted a network meta-analysis of first-linechemotherapy for advanced BTC and found that the gemcitabine + S-1 regimenwas superior to gemcitabine monotherapy in OS(HR = 0.43, 95%CI0.20 - 0.93) and ORR(OR = 4.72, 95%CI 1.31 - 17.02) in patients with advancedbiliary cancer. Compared with 5-FU monotherapy, the ORRs of gemcitabine + S-1 (OR = 9.08, 95%CI 1.56 - 89.20) or capecitabine + cisplatin (OR = .46, 95%CI 1.07 - 56.63) were superior to 5-FU monotherapy. The OS ratesof gemcitabine + S-1 (HR = 0.43, 95%CI 0.20 - 0.93), gemcitabine +oxaliplatin (HR = 0.57, 95%CI 0.32 - 0.96), and fluorouracil + cisplatin (HR = 1.88,95%CI 1.07 - 3.16) were better than that of 5-FU monotherapy.

Yanfeng Jiang et al. [33] compared 20 chemotherapy or targeted therapy regimens of 24 studies related to advanced BTC and found that the FOLFOX-4(HR = 2.88, 95%CI 1.05—7.93), C-GEMOX(HR = 2.82, 95%CI 1.20—6.62), and GEMOX + erlotinib (HR = 3.21,95%CI 1.38—7.56) regimens had the most significant effect on prolonging PFS. Meanwhile, the ORR of the GEMOX + erlotinib regimen (OR = 0.13, 95%CI 0.02—0.87) was higher than that of 5-FU monotherapy.

##### Secondary outcomes

The incidence of neutropenia in the GP(OR = 0.06, 95%CI 0.01—0.50), G + S-1(OR = 0.05, 95%CI 0.00—0.55), C-GEMOX(OR = 0.08, 95%CI 0.01—0.60), RAM + GP(OR = 0.03, 95%CI 0.00—0.38) and MER + GP(OR = 0.03, 95%CI 0.00—0.41)regimens was lower than that in 5-FU monotherapy.

For the incidence of vomiting (OR = 0.07, 95%CI 0.00—0.98) and diarrhea (OR = 0.09, 95%CI 0.01—0.63), the XELOX chemotherapy regimen was lower than the G + XELOX regimen and GEMOX + erlotinib regimen.

## Discussion

### Summary of main results

Our main objectives were to provide an overview of the efficacy and safety of chemotherapy or targeted therapy for patients with advanced BTC. Additionally, we aimed to review and identify inconsistencies in approaches adopted to evaluate the evidence in published reviews. We planned to use this information to propose some strategies, which may effectively reduce the uncertainty in determining the effectiveness of systematic treatment for malignant biliary cancer. We mainly focused on the following comparison: (1) one combination chemotherapy versus another; (2) chemotherapy + targeted therapy versus chemotherapy; (3) combined chemotherapy versus single-drug chemotherapy; and (4) observation versus chemotherapy or targeted therapy.

Overall, we found that the quality of the reviews was not high, with 11 of the 14 reviews being “critically low” on the AMSTAR2 tool. We found two reviews that were assessed as “low,” and one review was assessed as “high.”

None of the reviews formally rated the evidence using the GRADE approach. We found most of the evidence within the included reviews to be of low quality. Overall, only 1 was rated as “High” quality, 10 were rated as “Moderate” quality, 27 were rated as “Low” quality, and 30 were rated as “Very Low.”

### Overall completeness and applicability of evidence

The GRADE approaches have defined the meaning of four evidence levels [42].

- High: We are certain that the real effect value is close to the estimated effect value, and further research is unlikely to change our confidence in the estimated effect value [42].
- Moderate: We have moderate confidence in the estimated value of the effect, and the real value may be close to the estimated value, but the two values may be quite different [42].
- Low: Our confidence in the estimated value of the effect is limited, and the actual value may be quite different from the estimated value [42].
- Very Low: We have little confidence in the estimated value of the effect. The real value is likely to be quite different from the estimated value, and any estimated value of the effect is very uncertain [42].

We classified the evidence with high or moderate level as “clear,” and the evidence with low or extremely low level was “unknown.” We comprehensively considered the evidence level and clinical outcome of all results and classified the evidence as follows:

- (1) clear evidence of benefit;
- (2) clear evidence of harm;
- (3) clear evidence of no effect or equivalence; and.
- (4) unknown benefit or harm or no effect or equivalence.

For evidence classified as “clear,” we have confidence in it to guide clinical treatment. However, more data are needed for further verification in the future. For “unknown” evidence, we do not recommend it as a basis for clinical diagnosis and treatment.

### Potential biases in the overview process

We are aware of the risk of introducing bias at all stages of the overview review process and taking measures to minimize it. First, the protocol of our study was registered on PROSPERO(CRD42022324548), and this study was reported according to the PRISMA-ScR checklist. This was an overview of the system review and meta-analysis, and the search was conducted across all years up to April 9, 2022, within Web of science, PubMed, and the Cochrane Database of Systematic Reviews. At the time when this umbrella review was completed, some potential reviews had not yet been completed. Therefore, the findings we have reported in this overview do not include the new study results from these reviews.

Of the 14 reviews, we found 13 published well-designed, comprehensive search strategies. Of these, 10 had no language restrictions in their searches, whereas four appeared to restrict searches to English [22, 26, 30, 32]. Of the 14 included studies, 11 used a satisfactory technique for assessing the risk of bias (RoB) in individual studies that were included in the review. Three studies did not report the quality of the individual studies they included, so we downgraded these studies in the final quality assessment. Two authors independently assessed these reviews for inclusion, carried out data collection, assessed the methodological quality of the included reviews via the ARMSTAR2 tool, and analyzed the quality of the evidence via GRADE approaches.

### Resolution of disagreements between evidence from the same topic

One of the clinical dilemmas that the umbrella review aims to solve is how to screen reliable evidence from massive evidence to guide clinical decision-making. In this umbrella review, we included some SRoMAs with the same research theme but opposite conclusions. Even for studies on the same topic, the number and quality of the original studies they included varied, and there were also differences in the collection and integration of data across studies, which may partly explain the differences in conclusions between studies. For this reason, we used the GRADE approach to evaluate the quality of the relevant evidence in detail. In the context of the same clinical problem, we chose evidence of high-quality level as the preferred recommendation. For example, in this study, we compared the safety of gemcitabine-based chemotherapy + targeted therapy with gemcitabine-based chemotherapy in patients with advanced biliary tract cancer. We found that the risk of neutropenia (RR [22] = 1.95, 95% CI 1.13–3.36, GRADE: Low; OR [21] = 1.37, 95% CI 0.89–2.12, GRADE: Very Low) and diarrhea (RR [22] = 1.65, 95% CI 0.89–3.04, GRADE: Very Low; RR [17] = 2.48, 95% CI 1.2–5.10, GRADE: Moderate) was different in various studies [17, 21, 22]. After GRADE assessment, we have more reason to believe in evidence with a high quality of evidence. Therefore, patients receiving chemotherapy + targeted therapy have a higher risk of neutropenia and diarrhea than patients receiving gemcitabine-based chemotherapy.

### Strengths and limitations

Umbrella review is a new method of evidence-based medicine analysis and is the highest level of evidence in the field of evidence-based medicine. Umbrella review is based on and superior to systematic review and meta-analysis. It is a comprehensive review of all systematic reviews and meta-analyses published to date on a particular medical topic, with consequent analysis of the level of quality of evidence [4, 43]. In the past decade, the number of systematic review and meta-analysis studies has increased significantly, largely addressing the lack of evidence in clinical decision-making. However, currently, there is too much evidence about the same medical problem, and the quality of evidence is uneven, which brings difficulties for doctors to make clinical decisions. Therefore, umbrella review plays an increasingly prominent role in evidence-based medicine and is attracting rising attention by clinicians.

For this study, first, we used an umbrella review approach to review almost all current evidence for the systemic treatment of biliary malignancies, which is the latest and most comprehensive collection of evidence to date. Second, we systematically divided the levels of evidence according to GRADE, so that clinicians can intuitively understand the authenticity and clinical applicability of different forms of evidence. Third, we discussed some evidence with the same theme but opposite conclusions, which partly solved the difficulties in clinical decision-making caused by mixed evidence.

However, possible limitations should be considered in the interpretation of this topic. First, our study included only a systematic review and meta-analysis, and it did not include original studies such as randomized controlled studies or retrospective studies. This may have kept us from examining recent advances in chemotherapy or targeted therapies for biliary cancer. Second, for some of the SRoMAs we included, we were unable to conduct excessive significance tests due to the lack of original data. Third, some of the included meta-analyses were not tested for publication bias, which may lead to potential publication bias. Finally, because the evidence summary table (NMA-SoF) for continuous variables of the network meta-analysis had not been tested by the GRADE working group, we did not evaluate the quality of evidence for the network meta-analyses in this study.

## Conclusions

This study comprehensively evaluated the safety and efficacy of chemotherapy or targeted therapy regimens for advanced biliary tract cancer. We found 11 “moderate” or “high” levels of evidence; however, most of the evidence was still at “low” or “very low” levels. Overall, there is still a lack of high-quality evidence on the effect of different chemotherapy or targeted therapy regimens on patient survival, and more randomized controlled studies are needed in the future to further summarize high levels of evidence.

## Supplementary Information

**Additional file 1.**

**Additional file 2.**

**Additional file 3**.

**Additional file 4**.

## Data Availability

All data generated or analysed during this study are included in this published article.

## Abbreviations

5-FU/LV: 5-Fluorouracil + Leucovorin
ASC: Active Symptom Control
Cap: Capecitabine
CapC: Capecitabine + Ciaplatin
C-GEMOX: Cetuximab + GEMOX
CHT: Chemotherapy
DCR: Disease Control Rate
DRR: Disease response rate
EGFR: Epidermal Growth Factor Receptor
FOLFOX-4: 5-FU + Folinic acid + Oxaliplatin
FP: Fluoropyrimidine(5-FU, capecitabine, or S-1) + cisplatin
G/Gem: Gemcitabine
GC: Gemcitabine + carboplatin
GEMOX: Gemcitabine + Oxaliplatin
GP: Gemcitabine + cisplatin
GS: Gemcitabine + S-1
HR: Hazard Ratio
mFOLFOX: Oxaliplatin + 5-FU
MER: Merestinib
OIS: Optimal information size
OR: Odds Ratio
ORR: Overall Response Rate
OS: Overall Survival
OX: Oxaliplatin
PFS: Progression-Free Survival
RAM: Ramucirumab
RCTs: Randomized controlled trials
RR: Relative Risk
S: S = S-1
SRoMAs: Systematic reviews and meta-analyses
SP: S-1 + Cisplatin
TAs: Targeted agents
TTP: Time to Progression
VEGFR: Vascular Endothelial Growth Factor Receptor
WMD: Weighted Mean Difference
XELOX: Capecitabine + Oxaliplatin
XP: Capecitabine + cisplatin
