# Supplementary material for: Chemotherapy and targeted therapy for advanced biliary tract cancers: an umbrella review
Source: BMC Cancer. 2023 Apr 25;23:378. doi: 10.1186/s12885-023-10679-8 (PMC10131316; doi:10.1186/s12885-023-10679-8)
Supplement: Supplementary file 2 — Additional file 2. [file 12885_2023_10679_MOESM2_ESM.pdf]

Table S2. Search terms utilized in the umbrella review.(Search date up to 09 April, 2022)

**Datebases searches**				
**Terms 1**	**Terms 2**	**Database**	**Restrictions**	**Results**
"gallbladder cancer" OR "gallbladder carcinoma" OR "gallbladder neoplasms" OR "gallbladder tumor" OR "gallbladder neoplasm" OR "gallbladder mass" OR "gallbladder masses" OR "biliary Cancer" OR "biliary tumor" OR "biliary neoplasms" OR "biliary neoplasm" OR "biliary mass" OR "biliary masses" OR "cholangiocarcinoma" OR "bile duct cancer" OR "bile duct tumor" OR "bile duct neoplasms" OR "bile duct neoplasm" OR "bile duct mass" OR "bile duct masses"	meta-analysis OR systematic review OR systematic overview	PubMed	All field	1268
TS=(gallbladder cancer OR gallbladder carcinoma OR gallbladder neoplasms OR gallbladder tumor OR gallbladder neoplasm OR gallbladder mass OR gallbladder masses OR biliary Cancer OR biliary tumor OR biliary neoplasms OR biliary neoplasm OR biliary mass OR biliary masses OR cholangiocarcinoma OR bile duct cancer OR bile duct tumor OR bile duct neoplasms OR bile duct neoplasm OR bile duct mass OR bile duct masses)	TS=(meta-analysis OR systematic review OR systematic overview)	Web Of Science	Topic	1332

gallbladder cancer OR gallbladder carcinoma OR gallbladder neoplasms OR gallbladder tumor OR gallbladder neoplasm OR gallbladder mass OR gallbladder masses OR biliary Cancer OR biliary tumor OR biliary neoplasms OR biliary neoplasm OR biliary mass OR biliary masses OR cholangiocarcinoma OR bile duct cancer OR bile duct tumor OR bile duct neoplasms OR bile duct neoplasm OR bile duct mass OR bile duct masses	meta-analysis OR systematic review OR systematic overview	Cochrane database of Systematic Reviews	Cochrane Reviews	52
Manual searches				
[1] Ying J, Chen J. Combination versus mono-therapy as salvage treatment for advanced biliary tract cancer: A comprehensive meta-analysis of published data. Crit Rev Oncol Hematol. 2019. 139: 134-142.				
[2] Jiang Q, Huang J, Zhang B, et al. Efficacy and Safety of Anti-PD1/PDL1 in Advanced Biliary Tract Cancer: A Systematic Review and Meta-Analysis. Front Immunol. 2022. 13: 801909.				
