# Supplementary material for: Chemotherapy and targeted therapy for advanced biliary tract cancers: an umbrella review
Source: BMC Cancer. 2023 Apr 25;23:378. doi: 10.1186/s12885-023-10679-8 (PMC10131316; doi:10.1186/s12885-023-10679-8)
Supplement: Supplementary file 3 — Additional file 3. [file 12885_2023_10679_MOESM3_ESM.pdf]

Table S3:Reasons for literature exclusion in full-text screening

Exclusion reason	Reference number
1.Not systematic review or meta analysis.	[1]
2.Meta analysis of multiple tumors.	[2]
3.Adjuvant therapy is radiotherapy or local treatment.	[3-6]
4.No comparison between chemotherapy regimens.	[7-18]
5.Data overlapped.	[19-23]
6.OR/ HR / RR values were not calculated.	[24-27]

Reference List

[1] Kamarajah S, Giovinazzo F, Roberts KJ, et al. The role of down staging treatment in the management of locally advanced intrahepatic cholangiocarcinoma: Review of literature and pooled analysis. Ann Hepatobiliary Pancreat Surg. 2020. 24(1): 6-16.

[2] Qi WX, Tang LN, He AN, Yao Y, Shen Z. Incidence and risk of treatment-related mortality in cancer patients treated with EGFR-TKIs: a meta-analysis of 22 phase III randomized controlled trials. Respir Med. 2013. 107(8): 1280-3.

[3] Hakeem AR, Papoulas M, Menon KV. The role of neoadjuvant chemotherapy or chemoradiotherapy for advanced gallbladder cancer - A systematic review. Eur J Surg Oncol. 2019. 45(2): 83-91.

[4] Chen X, Meng F, Xiong H, Zou Y. Adjuvant Therapy for Resectable Biliary Tract Cancer: A Bayesian Network Analysis. Front Oncol. 2021. 11: 600027.

[5] Ray CE Jr, Edwards A, Smith MT, et al. Metaanalysis of survival, complications, and imaging response following chemotherapy-based transarterial therapy in patients with unresectable intrahepatic cholangiocarcinoma. J Vasc Interv Radiol. 2013. 24(8): 1218-26.

[6] Holster JJ, El Hassnaoui M, Franssen S, et al. Hepatic Arterial Infusion Pump Chemotherapy for Unresectable Intrahepatic Cholangiocarcinoma: A Systematic Review and Meta-Analysis. Ann Surg Oncol. 2022 .

[7] Luvira V, Satitkarnmanee E, Pugkhem A, Kietpeerakool C, Lumbiganon P, Pattanittum P. Postoperative adjuvant chemotherapy for resectable cholangiocarcinoma. Cochrane Database Syst Rev. 2021. 9: CD012814.

[8] Moriwaki T, Yamamoto Y, Gosho M, et al. Correlations of survival with progression-free survival, response rate, and disease control rate in advanced biliary tract cancer: a meta-analysis of randomised trials of first-line chemotherapy. Br J Cancer. 2016. 114(8): 881-8.

[9] Messina C, Merz V, Frisinghelli M, et al. Adjuvant chemotherapy in resected bile duct cancer: A systematic review and meta-analysis of randomized trials. Crit Rev Oncol Hematol. 2019. 143: 124-129.

[10] Ghidini M, Tomasello G, Botticelli A, et al. Adjuvant chemotherapy for resected biliary tract cancers: a systematic review and meta-analysis. HPB (Oxford). 2017. 19(9): 741-748.

[11] Grendar J, Grendarova P, Sinha R, Dixon E. Neoadjuvant therapy for

downstaging of locally advanced hilar cholangiocarcinoma: a systematic review. HPB (Oxford). 2014. 16(4): 297-303.
[12] Lamarca A, Hubner RA, David Ryder W, Valle JW. Second-line chemotherapy in advanced biliary cancer: a systematic review. Ann Oncol. 2014. 25(12): 2328-2338.
[13] Rangarajan K, Simmons G, Manas D, Malik H, Hamady ZZ. Systemic adjuvant chemotherapy for cholangiocarcinoma surgery: A systematic review and meta-analysis. Eur J Surg Oncol. 2020. 46(4 Pt A): 684-693.
[14] Caparica R, Bruzzone M, Hachem GE, et al. Adjuvant chemotherapy in biliary tract cancer patients: A systematic review and meta-analysis of randomized controlled trials. Crit Rev Oncol Hematol. 2020. 149: 102940.
[15] Azizi AA, Lamarca A, McNamara MG, Valle JW. Chemotherapy for advanced gallbladder cancer (GBC): A systematic review and meta-analysis. Crit Rev Oncol Hematol. 2021. 163: 103328.
[16] Ma KW, Cheung TT, Leung B, et al. Adjuvant chemotherapy improves oncological outcomes of resectable intrahepatic cholangiocarcinoma: A meta-analysis. Medicine (Baltimore). 2019. 98(5): e14013.
[17] Belkouz A, Nooijen LE, Riady H, et al. Efficacy and safety of systemic induction therapy in initially unresectable locally advanced intrahepatic and perihilar cholangiocarcinoma: A systematic review. Cancer Treat Rev. 2020. 91: 102110.
[18] Song S, Yang W, Tian H, et al. The efficacy and safety of 5-fluorouracil based adjuvant therapy in resected biliary tract cancer: A systematic review and meta-analysis. Clin Res Hepatol Gastroenterol. 2022. 46(2): 101788.
[19] Sun XF, He ZK, Sun JP, Ge QX, Shen ED. The efficacy and safety of different pharmacological interventions for patients with advanced biliary tract cancer: A network meta-analysis. Oncotarget. 2017. 8(59): 100657-100667.
[20] Yang R, Wang B, Chen YJ, Li HB, Hu JB, Zou SQ. Efficacy of gemcitabine plus platinum agents for biliary tract cancers: a meta-analysis. Anticancer Drugs. 2013. 24(8): 871-7.
[21] Cai W, Yuan Y, Ge W, et al. EGFR Target Therapy Combined with Gemox for Advanced Biliary Tract Cancers: a Meta-analysis based on RCTs. J Cancer. 2018. 9(8): 1476-1485.
[22] Valle JW, Furuse J, Jitlal M, et al. Cisplatin and gemcitabine for advanced biliary tract cancer: a meta-analysis of two randomised trials. Ann Oncol. 2014. 25(2): 391-8.
[23] Kish M, Chan K, Perry K, Ko YJ. A systematic review and network meta-analysis of adjuvant therapy for curatively resected biliary tract cancers. Curr Oncol. 2020. 27(1): e20-e26.
[24] Naveed S, Qari H, Thau CM, Burasakarn P, Mir AW. Neoadjuvant Chemotherapy for Advanced Gallbladder Cancer: Do We have Enough Evidence? A Systematic Review. Euroasian J Hepatogastroenterol. 2021. 11(2): 87-94.
[25] Fiteni F, Nguyen T, Vernerey D, et al. Cisplatin/gemcitabine or oxaliplatin/gemcitabine in the treatment of advanced biliary tract cancer: a

systematic review. Cancer Med. 2014. 3(6): 1502-11.
[26] Jiang Q, Huang J, Zhang B, et al. Efficacy and Safety of Anti-PD1/PDL1 in Advanced Biliary Tract Cancer: A Systematic Review and Meta-Analysis. Front Immunol. 2022. 13: 801909.
[27] Park JO, Oh DY, Hsu C, et al. Gemcitabine Plus Cisplatin for Advanced Biliary Tract Cancer: A Systematic Review. Cancer Res Treat. 2015. 47(3): 343-61.
