# Supplementary material for: Chemotherapy and targeted therapy for advanced biliary tract cancers: an umbrella review
Source: BMC Cancer. 2023 Apr 25;23:378. doi: 10.1186/s12885-023-10679-8 (PMC10131316; doi:10.1186/s12885-023-10679-8)
Supplement: Supplementary file 4 — Additional file 4. [file 12885_2023_10679_MOESM4_ESM.pdf]

Author(s):
Question: Gem-based+ anti-EGFR compared to Gem-based for Survival and Toxicity
Setting:
Bibliography:

Certainty assessment							№ of patients		Effect		Certainty	Importance
**№ of studies**	**Study design**	**Risk of bias**	**Inconsistency**	**Indirectness**	**Imprecision**	**Other considerations**	**Gem-based+ anti-EGFR**	**Gem-based**	**Relative (95% CI)**	**Absolute (95% CI)**		
**OS**												
4	randomised trials	serious	not serious	not serious	not serious	publication bias strongly suspected[a]	228 participants	222 participants	**HR 0.82** (0.64 to 1.06) [OS]	**-- per 1,000** (from -- to --)	⊕⊕◯◯ Low	CRITICAL
							-	0.0%		**-- per 1,000** (from -- to --)		
**PFS**												
4	randomised trials	serious	not serious	not serious	not serious	publication bias strongly suspected[a]	228 participants	222 participants	**HR 0.88** (0.73 to 1.08) [DFS]	**-- per 1,000** (from -- to --)	⊕⊕◯◯ Low	CRITICAL
							-	0.0%		**-- per 1,000** (from -- to --)		
**ORR**												
3	randomised trials	serious	not serious	not serious	serious[b]	publication bias strongly suspected[a]	47/183 (25.7%)	34/178 (19.1%)	**RR 1.34** (0.91 to 1.99)	**65 more per 1,000** (from 17 fewer to 189 more)	⊕◯◯◯ Very low	CRITICAL
**Toxicities--Neutropenia**												
4	randomised trials	serious	not serious	not serious	not serious	publication bias strongly suspected[a]	34/228 (14.9%)	17/222 (7.7%)	**RR 1.95** (1.13 to 3.36)	**73 more per 1,000** (from 10 more to 181 more)	⊕⊕◯◯ Low	IMPORTANT
**Toxicities--Thrombocytopenia**												
4	randomised trials	serious	not serious	not serious	serious[b]	publication bias strongly suspected[a]	28/228 (12.3%)	16/222 (7.2%)	**RR 1.69** (0.99 to 2.87)	**50 more per 1,000** (from 1 fewer to 135 more)	⊕◯◯◯ Very low	IMPORTANT
**Toxicities--Skin rash**												
4	randomised trials	serious	not serious	not serious	serious[c]	publication bias strongly suspected very strong association[a]	45/228 (19.7%)	1/222 (0.5%)	**RR 18.11** (5.13 to 63.91)	**77 more per 1,000** (from 19 more to 283 more)	⊕⊕⊕◯ Moderate	IMPORTANT
**Toxicities--Diarrhea**												
4	randomised trials	serious	not serious	not serious	serious[b]	publication bias strongly suspected[a]	24/228 (10.5%)	14/222 (6.3%)	**RR 1.65** (0.89 to 3.04)	**41 more per 1,000** (from 7 fewer to 129 more)	⊕◯◯◯ Very low	IMPORTANT
**Toxicities--Fatigue**												
4	randomised trials	serious	not serious	not serious	serious[b]	publication bias strongly suspected[a]	17/228 (7.5%)	8/222 (3.6%)	**RR 2.01** (0.91 to 4.44)	**36 more per 1,000** (from 3 fewer to 124 more)	⊕◯◯◯ Very low	IMPORTANT

CI: confidence interval; **HR:** hazard Ratio; **RR:** risk ratio

Explanations

a. No publication bias test was performed
b. Sample size less than OIS
c. The range of confidence interval is too large

Author(s):
Question: GP compared to G for Survival
Setting:
Bibliography:

Certainty assessment							№ of patients		Effect		Certainty	Importance
**№ of studies**	**Study design**	**Risk of bias**	**Inconsistency**	**Indirectness**	**Imprecision**	**Other considerations**	**GP**	**G**	**Relative (95% CI)**	**Absolute (95% CI)**		
**OS (assessed with: MD)**												
2	randomised trials	serious	not serious	not serious	not serious	publication bias strongly suspected[a]	202	184	-	MD **3.52 fewer** (5.14 fewer to 1.35 fewer)	⨁⨁◯◯ Low	CRITICAL
**OS (assessed with: HR)**												
2	randomised trials	serious	not serious	not serious	not serious	publication bias strongly suspected[a]	202 participants	184 participants	**HR 0.65** (0.53 to 0.79) [OS]	**-- per 1,000** (from -- to --)	⨁⨁◯◯ Low	CRITICAL
							-	0.0%		**-- per 1,000** (from -- to --)		
**PFS**												
2	randomised trials	serious	not serious	not serious	not serious	publication bias strongly suspected[a]	202	184	-	MD **2.6 lower** (3.81 lower to 1.4 lower)	⨁⨁◯◯ Low	CRITICAL
**PFS**												
2	randomised trials	serious	not serious	not serious	not serious	publication bias strongly suspected[a]	202 participants	184 participants	**HR 0.63** (0.52 to 0.76) [PFS]	**-- per 1,000** (from -- to --)	⨁⨁◯◯ Low	CRITICAL
							-	0.0%		**-- per 1,000** (from -- to --)		
**ORR**												
2	randomised trials	serious	not serious	not serious	not serious	publication bias strongly suspected[a]	27/184 (14.7%)	50/202 (24.8%)	**OR 0.53** (0.31 to 0.88)	**99 fewer per 1,000** (from 155 fewer to 23 fewer)	⨁⨁◯◯ Low	CRITICAL

CI: confidence interval; **HR:** hazard Ratio; **MD:** mean difference; **OR:** odds ratio

Explanations

a. No publication bias test was performed

Author(s):
Question: GP+anti-EGFR compared to GP for Survival
Setting:
Bibliography:

Certainty assessment							№ of patients		Effect		Certainty	Importance
№ of studies	Study design	Risk of bias	Inconsistency	Indirectness	Imprecision	Other considerations	GP+anti-EGFR	GP	Relative (95% CI)	Absolute (95% CI)		
**OS**												
4	randomised trials	serious	not serious	not serious	not serious	publication bias strongly suspected[a]	316	313	-	MD **1.49 lower** (2.56 lower to 0.43 lower)	⨁⨁◯◯ Low	CRITICAL
**OS**												
4	randomised trials	serious	not serious	not serious	not serious	publication bias strongly suspected[a]	316 participants	313 participants	**HR 0.90** (0.70 to 1.15) [OS]	**-- per 1,000** (from -- to --)	⨁⨁◯◯ Low	CRITICAL
							-	0.0%		**-- per 1,000** (from -- to --)		
**PFS**												
4	randomised trials	serious	not serious	not serious	not serious	publication bias strongly suspected[a]	316	313	-	MD **0.07 lower** (1.91 lower to 1.77 higher)	⨁⨁◯◯ Low	CRITICAL
**PFS**												
4	randomised trials	serious	not serious	not serious	not serious	publication bias strongly suspected[a]	316 participants	313 participants	**HR 0.79** (0.63 to 0.99) [PFS]	**-- per 1,000** (from -- to --)	⨁⨁◯◯ Low	CRITICAL
							-	0.0%		**-- per 1,000** (from -- to --)		
**ORR**												
4	randomised trials	serious	not serious	not serious	not serious	publication bias strongly suspected[a]	87/316 (27.5%)	55/313 (17.6%)	**OR 0.56** (0.38 to 0.83)	**69 fewer per 1,000** (from 101 fewer to 25 fewer)	⨁⨁◯◯ Low	CRITICAL

CI: confidence interval; **HR:** hazard Ratio; **MD:** mean difference; **OR:** odds ratio

Explanations

a. No publication bias test was performed

Author(s):
Question: FP compared to GP for Survival and Toxicity
Setting:
Bibliography:

Certainty assessment							№ of patients		Effect		Certainty	Importance
**№ of studies**	**Study design**	**Risk of bias**	**Inconsistency**	**Indirectness**	**Imprecision**	**Other considerations**	**FP**	**GP**	**Relative (95% CI)**	**Absolute (95% CI)**		
**ORR**												
5	observational studies	serious	not serious	not serious	not serious	none	-/311	-/416	**RR 1.13** (0.80 to 1.58)	**0 fewer per 1,000** (from 0 fewer to 0 fewer)	⊕◯◯◯ Very low	CRITICAL
**DCR**												
5	observational studies	serious	not serious	not serious	not serious	none	-/311	-/416	**RR 1.02** (0.91 to 1.13)	**0 fewer per 1,000** (from 0 fewer to 0 fewer)	⊕◯◯◯ Very low	CRITICAL
**PFS**												
4	non-randomised studies	serious	not serious	not serious	not serious	publication bias strongly suspected[a]	233 participants<br>- 	360 participants<br>0.0%	**HR 0.95** (0.86 to 1.05) [PFS]	**-- per 1,000** (from -- to --)<br>**-- per 1,000** (from -- to --)	⊕⊕◯◯ Low	CRITICAL
**OS**												
4	non-randomised studies	serious	not serious	not serious	not serious	publication bias strongly suspected[a]	233 participants<br>-	360 participants<br>0.0%	**HR 1.06** (0.98 to 1.14) [OS]	**-- per 1,000** (from -- to --)<br>**-- per 1,000** (from -- to --)	⊕⊕◯◯ Low	CRITICAL
**Toxicities--Neutropenia**												
4	observational studies	serious	serious[b]	not serious	not serious	publication bias strongly suspected[a]	-/233	-/360	not estimable		⊕◯◯◯ Very low	IMPORTANT
**Toxicities--Anemia**												
4	observational studies	serious	serious[b]	not serious	not serious	publication bias strongly suspected[a]	-/233	-/360	not estimable		⊕◯◯◯ Very low	IMPORTANT
**Toxicities--Trombocytopenia**												
4	observational studies	serious	serious[b]	not serious	not serious	publication bias strongly suspected[a]	-/233	-/360	not estimable		⊕◯◯◯ Very low	IMPORTANT
**Toxicities--Nausea/Vomiting**												
4	observational studies	serious	not serious	not serious	not serious	publication bias strongly suspected[a]	-/233	-/360	not estimable		⊕◯◯◯ Very low	IMPORTANT
**Toxicities--Anorexia**												
4	observational studies	serious	serious[b]	not serious	not serious	publication bias strongly suspected[a]	-/233	-/360	not estimable		⊕◯◯◯ Very low	IMPORTANT
**Toxicities--Nephropathy**												
4	observational studies	serious	serious[b]	not serious	not serious	publication bias strongly suspected[a]	-/233	-/360	not estimable		⊕◯◯◯ Very low	IMPORTANT
**Toxicities--Neuropathy**												
4	observational studies	serious	serious[b]	not serious	not serious	publication bias strongly suspected[a]	-/233	-/360	not estimable		⊕◯◯◯ Very low	IMPORTANT

CI: confidence interval; **HR:** hazard Ratio; **RR:** risk ratio

Explanations

a. There was no publication bias test for this conclusion
b. The heterogeneity between the included studies was large

Author(s):
Question: G-based+anti-EGFR compared to G-based for Toxicities
Setting:
Bibliography:

Certainty assessment							№ of patients		Effect		Certainty	Importance
№ of studies	Study design	Risk of bias	Inconsistency	Indirectness	Imprecision	Other considerations	G-based+anti-EGFR	G-based	Relative (95% CI)	Absolute (95% CI)		
**Toxicities--Neutropenia**												
6	randomised trials	serious	not serious	not serious	serious[a]	publication bias strongly suspected[b]	62/429 (14.5%)	47/413 (11.4%)	**OR 1.37** (0.89 to 2.12)	**36 more per 1,000** (from 11 fewer to 100 more)	⨁◯◯◯ Very low	IMPORTANT
**Toxicities--Thrombocytopenia**												
6	randomised trials	serious	not serious	not serious	serious[a]	publication bias strongly suspected[b]	36/429 (8.4%)	25/413 (6.1%)	**OR 1.40** (0.83 to 2.39)	**22 more per 1,000** (from 10 fewer to 73 more)	⨁◯◯◯ Very low	IMPORTANT
**Toxicities--Anemia**												
4	randomised trials	serious	not serious	not serious	serious[a]	publication bias strongly suspected[b]	21/232 (9.1%)	17/223 (7.6%)	**OR 1.21** (0.62 to 2.38)	**15 more per 1,000** (from 28 fewer to 88 more)	⨁◯◯◯ Very low	IMPORTANT
**Toxicities--Peripheral neuropathy**												
5	randomised trials	serious	not serious	not serious	serious[a]	publication bias strongly suspected[b]	27/380 (7.1%)	17/365 (4.7%)	**OR 1.52** (0.81 to 2.88)	**23 more per 1,000** (from 9 fewer to 77 more)	⨁◯◯◯ Very low	IMPORTANT
**Toxicities--Increased AST/ALT**												
5	randomised trials	serious	not serious	not serious	serious[a]	publication bias strongly suspected[b]	37/380 (9.7%)	26/365 (7.1%)	**OR 1.65** (0.96 to 2.84)	**41 more per 1,000** (from 3 fewer to 108 more)	⨁◯◯◯ Very low	IMPORTANT

CI: confidence interval; **OR:** odds ratio

Explanations

a. Sample size less than OIS
b. No publication bias test was performed

Author(s):
Question: G-based compared to non-G-based for Survival and Toxicities
Setting:
Bibliography:

Certainty assessment							№ of patients		Effect		Certainty	Importance
№ of studies	Study design	Risk of bias	Inconsistency	Indirectness	Imprecision	Other considerations	G-based	non-G-based	Relative (95% CI)	Absolute (95% CI)		
**DRR**												
4	randomised trials	serious	not serious	not serious	serious[a]	none	139 participants	141 participants	**OR 1.39** (0.81 to 2.40) [DRR]	**0 fewer per 1,000** (from 0 fewer to 0 fewer)	⊕⊕◯◯ Low	CRITICAL
							-	0.0%		**0 fewer per 1,000** (from 0 fewer to 0 fewer)		
**DCR**												
4	randomised trials	serious	serious[b]	not serious	serious[a]	none	139 participants	141 participants	**OR 1.48** (0.43 to 5.07) [DCR]	**0 fewer per 1,000** (from 0 fewer to 0 fewer)	⊕◯◯◯ Very low	CRITICAL
							-	0.0%		**0 fewer per 1,000** (from 0 fewer to 0 fewer)		
**PFS**												
4	randomised trials	serious	serious[b]	not serious	not serious	none	139 participants	141 participants	not estimable		⊕⊕◯◯ Low	CRITICAL
							-	0.0%				
**OS**												
4	randomised trials	serious	serious[b]	not serious	not serious	none	139 participants	141 participants	not estimable		⊕⊕◯◯ Low	CRITICAL
							-	0.0%				
**Toxicities--Leukopenia**												
4	randomised trials	serious	serious[b]	not serious	not serious	publication bias strongly suspected very strong association[c]	41/148 (27.7%)	7/151 (4.6%)	**OR 7.17** (1.43 to 36.08)	**212 more per 1,000** (from 19 more to 591 more)	⊕⊕⊕◯ Moderate	IMPORTANT
**Toxicities--Anemia**												
3	randomised trials	serious	not serious	not serious	not serious	publication bias strongly suspected very strong association[c]	27/148 (18.2%)	5/151 (3.3%)	**OR 7.04** (2.59 to 19.12)	**161 more per 1,000** (from 48 more to 363 more)	⊕⊕⊕⊕ High	IMPORTANT
**Toxicities--Neutropenia**												
4	randomised trials	serious	serious[b]	not serious	serious[d]	publication bias strongly suspected[c]	68/148 (45.9%)	23/151 (15.2%)	**OR 4.63** (0.95 to 22.50)	**302 more per 1,000** (from 7 fewer to 649 more)	⊕◯◯◯ Very low	IMPORTANT
**Toxicities--Thrombocytopenia**												
4	randomised trials	serious	serious[b]	not serious	serious[d]	publication bias strongly suspected[c]	30/148 (20.3%)	13/151 (8.6%)	**OR 2.79** (0.66 to 11.81)	**122 more per 1,000** (from 28 fewer to 441 more)	⊕◯◯◯ Very low	IMPORTANT
**New outcome**												
4	randomised trials	serious	not serious	not serious	serious[a]	publication bias strongly suspected[c]	21/148 (14.2%)	20/151 (13.2%)	**OR 1.11** (0.56 to 2.23)	**12 more per 1,000** (from 54 fewer to 122 more)	⊕◯◯◯ Very low	IMPORTANT

CI: confidence interval; **OR:** odds ratio

Explanations

a. Sample size less than OIS
b. The heterogeneity between the included studies was large
c. There was no publication bias test for this conclusion
d. The sample size meets the OIS standard, but the 95% confidence interval contains invalid values

Author(s):
Question: G-based compared to G for Toxicities
Setting:
Bibliography:

Certainty assessment							№ of patients		Effect		Certainty	Importance
№ of studies	Study design	Risk of bias	Inconsistency	Indirectness	Imprecision	Other considerations	G-based	G	Relative (95% CI)	Absolute (95% CI)		
**Toxicities--Leukopenia**												
3	randomised trials	serious	not serious	not serious	not serious	publication bias strongly suspected[a]	53/269 (19.7%)	33/273 (12.1%)	**OR 1.82** (1.13 to 2.94)	**79 more per 1,000** (from 14 more to 167 more)	⊕⊕◯◯ Low	IMPORTANT
**Toxicities--Anemia**												
3	randomised trials	serious	serious[b]	not serious	not serious	publication bias strongly suspected[a]	33/269 (12.3%)	19/273 (7.0%)	**OR 1.96** (1.07 to 3.62)	**58 more per 1,000** (from 5 more to 143 more)	⊕◯◯◯ Very low	IMPORTANT
**Toxicities--Neutropenia**												
3	randomised trials	serious	not serious	not serious	not serious	publication bias strongly suspected[a]	83/269 (30.9%)	56/273 (20.5%)	**OR 1.78** (1.19 to 2.66)	**110 more per 1,000** (from 30 more to 202 more)	⊕⊕◯◯ Low	IMPORTANT
**Toxicities--Thrombocytopenia**												
3	randomised trials	serious	not serious	not serious	serious[c]	publication bias strongly suspected[a]	22/269 (8.2%)	20/273 (7.3%)	**OR 1.13** (0.60 to 2.14)	**9 more per 1,000** (from 28 fewer to 71 more)	⊕◯◯◯ Very low	IMPORTANT
**Toxicities-- Increased ALT level**												
3	randomised trials	serious	serious[b]	not serious	serious[c]	publication bias strongly suspected[a]	32/269 (11.9%)	41/273 (15.0%)	**OR 0.76** (0.47 to 1.25)	**32 fewer per 1,000** (from 73 fewer to 31 more)	⊕◯◯◯ Very low	IMPORTANT

CI: confidence interval; **OR:** odds ratio

Explanations

a. There was no publication bias test for this conclusion
b. The heterogeneity between the included studies was large
c. Sample size less than OIS

Author(s):
Question: G-based+anti-VEGFR/EGFR compared to G-based for Toxicities
Setting:
Bibliography:

Certainty assessment							№ of patients		Effect		Certainty	Importance
№ of studies	Study design	Risk of bias	Inconsistency	Indirectness	Imprecision	Other considerations	G-based+anti-VEGFR/EGFR	G-based	Relative (95% CI)	Absolute (95% CI)		
**Toxicities-- Nausea**												
6	randomised trials	serious	not serious	not serious	serious[a]	publication bias strongly suspected[b]	12/408 (2.9%)	12/403 (3.0%)	**RR 1.01** (0.41 to 2.47)	**0 fewer per 1,000** (from 18 fewer to 44 more)	⨁◯◯◯ Very low	IMPORTANT
**Toxicities-- Vomiting**												
6	randomised trials	serious	not serious	not serious	serious[a]	publication bias strongly suspected[b]	11/346 (3.2%)	17/343 (5.0%)	**RR 0.71** (0.31 to 1.60)	**14 fewer per 1,000** (from 34 fewer to 30 more)	⨁◯◯◯ Very low	IMPORTANT
**Toxicities-- Diarrhea**												
6	randomised trials	serious	not serious	not serious	not serious	publication bias strongly suspected strong association[b]	28/408 (6.9%)	11/403 (2.7%)	**RR 2.48** (1.20 to 5.10)	**40 more per 1,000** (from 5 more to 112 more)	⨁⨁⨁◯ Moderate	IMPORTANT

CI: confidence interval; **RR:** risk ratio

Explanations

a. Sample size less than OIS
b. There was no publication bias test for this conclusion

Author(s):
Question: Fluoropyrimidine-based doublet CHT compared to ASC or 5-FU/LV for Survival
Setting:
Bibliography:

Certainty assessment							№ of patients		Effect		Certainty	Importance
№ of studies	Study design	Risk of bias	Inconsistency	Indirectness	Imprecision	Other considerations	Fluoropyrimidine-based doublet CHT	ASC or 5-FU/LV	Relative (95% CI)	Absolute (95% CI)		
**OS**												
2	randomised trials	serious	not serious	not serious	not serious	publication bias strongly suspected[a]	169 participants	167 participants	**HR 0.63** (0.49 to 0.80) [OS]	**-- per 1,000** (from -- to --)	⨁⨁◯◯ Low	
							-	0.0%		**-- per 1,000** (from -- to --)		
**DCR**												
2	randomised trials	serious	serious[b]	not serious	not serious	publication bias strongly suspected very strong association[a]	169 participants	167 participants	**OR 13.29** (0.39 to 456.18) [DCR]	**0 fewer per 1,000** (from 0 fewer to 0 fewer)	⨁⨁⨁◯ Moderate	
							-	0.0%		**0 fewer per 1,000** (from 0 fewer to 0 fewer)		
							-	34.9%		**528 more per 1,000** (from 176 fewer to 647 more)		
**ORR**												
2	randomised trials	serious	not serious	not serious	not serious	publication bias strongly suspected strong association[a]	169 participants	167 participants	**OR 3.24** (1.18 to 8.92) [ORR]	**0 fewer per 1,000** (from 0 fewer to 0 fewer)	⨁⨁⨁◯ Moderate	
							-	0.0%		**0 fewer per 1,000** (from 0 fewer to 0 fewer)		
							-	5.8%		**108 more per 1,000** (from 10 more to 297 more)		

CI: confidence interval; **HR:** hazard Ratio; **OR:** odds ratio

Explanations

a. There was no publication bias test for this conclusion
b. The heterogeneity between the included studies was large

Author(s):
Question: Fluoropyrimidine-based compared to Observation for Survival
Setting:
Bibliography:

Certainty assessment							№ of patients		Effect		Certainty	Importance
№ of studies	Study design	Risk of bias	Inconsistency	Indirectness	Imprecision	Other considerations	Fluoropyrimidine-based	Observation	Relative (95% CI)	Absolute (95% CI)		
**OS**												
4	randomised trials	serious	not serious	not serious	not serious	none	381 participants	358 participants	**HR 0.83** (0.70 to 0.99) [OS]	**-- per 1,000** (from -- to --)	⨁⨁⨁◯ Moderate	IMPORTANT
							-	0.0%		**-- per 1,000** (from -- to --)		

CI: confidence interval; **HR:** hazard Ratio

Author(s):
Question: G-based compared to Observation for Survival
Setting:
Bibliography:

Certainty assessment							№ of patients		Effect		Certainty	Importance
№ of studies	Study design	Risk of bias	Inconsistency	Indirectness	Imprecision	Other considerations	G-based	Observation	Relative (95% CI)	Absolute (95% CI)		
**OS**												
3	randomised trials	serious	not serious	not serious	not serious	none	246 participants	238 participants	**HR 0.91** (0.74 to 1.12) [OS]	**-- per 1,000** (from -- to --)	⨁⨁⨁◯ Moderate	IMPORTANT
							-	0.0%		**-- per 1,000** (from -- to --)		

CI: confidence interval; **HR:** hazard Ratio

Author(s):
Question: G-based compared to Observation for Survival
Setting:
Bibliography:

Certainty assessment							№ of patients		Effect		Certainty	Importance
**№ of studies**	**Study design**	**Risk of bias**	**Inconsistency**	**Indirectness**	**Imprecision**	**Other considerations**	**G-based**	**Observation**	**Relative (95% CI)**	**Absolute (95% CI)**		
**RFS-All Patients**												
2	randomised trials	serious	not serious	not serious	not serious	publication bias strongly suspected[a]	212 participants	207 participants	**HR 0.91** (0.71 to 1.16) [RFS-All Patients]	**-- per 1,000** (from -- to --)	⨁⨁◯◯ Low	CRITICAL
							-	0.0%		**-- per 1,000** (from -- to --)		
**RFS-R1 resection Patients**												
2	randomised trials	serious	not serious	not serious	not serious	publication bias strongly suspected[a]	212 participants	207 participants	**HR 1.10** (0.58 to 2.07) [RFS-R1 resection Patients]	**-- per 1,000** (from -- to --)	⨁⨁◯◯ Low	CRITICAL
							-	0.0%		**-- per 1,000** (from -- to --)		
**RFS-N+ tumor Patients**												
2	randomised trials	serious	not serious	not serious	not serious	publication bias strongly suspected[a]	212 participants	207 participants	**HR 0.86** (0.60 to 1.23) [RFS-N+ tumor Patients]	**-- per 1,000** (from -- to --)	⨁⨁◯◯ Low	CRITICAL
							-	0.0%		**-- per 1,000** (from -- to --)		
**OS-All Patients**												
2	randomised trials	serious	not serious	not serious	not serious	publication bias strongly suspected[a]	212 participants	207 participants	**HR 1.03** (0.78 to 1.35) [OS-All Patients]	**-- per 1,000** (from -- to --)	⨁⨁◯◯ Low	CRITICAL
							-	0.0%		**-- per 1,000** (from -- to --)		
**OS-R1 resection Patients**												
2	randomised trials	serious	not serious	not serious	not serious	publication bias strongly suspected[a]	212 participants	207 participants	**HR 1.25** (0.63 to 2.49) [OS-R1 resection Patients]	**-- per 1,000** (from -- to --)	⨁⨁◯◯ Low	CRITICAL
							-	0.0%		**-- per 1,000** (from -- to --)		
**OS-N+ tumor Patients**												
2	randomised trials	serious	not serious	not serious	not serious	publication bias strongly suspected[a]	212 participants	207 participants	**HR 0.99** (0.67 to 1.46) [OS-N+ tumor Patients]	**-- per 1,000** (from -- to --)	⨁⨁◯◯ Low	CRITICAL
							-	0.0%		**-- per 1,000** (from -- to --)		

CI: confidence interval; **HR:** hazard Ratio

Explanations

a. There was no publication bias test for this conclusion

Author(s):
Question: G+S-1 compared to S-1 for Survival and Toxicities
Setting:
Bibliography:

Certainty assessment							№ of patients		Effect		Certainty	Importance
**№ of studies**	**Study design**	**Risk of bias**	**Inconsistency**	**Indirectness**	**Imprecision**	**Other considerations**	**G+S-1**	**S-1**	**Relative (95% CI)**	**Absolute (95% CI)**		
**All-cause mortality at 1 year**												
2	randomised trials	serious	serious[a]	not serious	not serious	publication bias strongly suspected[b]	76 participants	75 participants	**RR 0.61** (0.33 to 1.13) [All-cause mortality at 1 year]	**0 fewer per 1,000** (from 0 fewer to 0 fewer)	⊕◯◯◯ Very low	
							-	0.0%		**0 fewer per 1,000** (from 0 fewer to 0 fewer)		
**ORR(S-I vs.G+S-1)**												
2	randomised trials	serious	not serious	not serious	not serious	publication bias strongly suspected strong association[b]	69 participants	71 participants	**RR 2.46** (1.27 to 4.75) [ORR]	**0 fewer per 1,000** (from 0 fewer to 0 fewer)	⊕⊕⊕◯ Moderate	
							-	0.0%		**0 fewer per 1,000** (from 0 fewer to 0 fewer)		
**Toxicities--Grade 1 - 4 Anaemia**												
2	randomised trials	serious	not serious	not serious	serious[c]	publication bias strongly suspected[b]	47/76 (61.8%)	36/75 (48.0%)	**RR 1.26** (1.00 to 1.59)	**125 more per 1,000** (from 0 fewer to 283 more)	⊕◯◯◯ Very low	
**Toxicities--Grade 1 - 4 Thrombocytopenia**												
2	randomised trials	serious	not serious	not serious	not serious	publication bias strongly suspected strong association[b]	31/76 (40.8%)	12/75 (16.0%)	**RR 2.45** (1.39 to 4.32)	**232 more per 1,000** (from 62 more to 531 more)	⊕⊕⊕◯ Moderate	
**Toxicities--Grade 1 - 4 Neutropenia**												
2	randomised trials	serious	not serious	not serious	not serious	publication bias strongly suspected strong association[b]	59/76 (77.6%)	22/75 (29.3%)	**RR 3.30** (1.04 to 10.50)	**675 more per 1,000** (from 12 more to 1,000 more)	⊕⊕⊕◯ Moderate	
**Toxicities--Febrile Neutropenia**												
2	randomised trials	serious	not serious	not serious	very serious[c,d]	publication bias strongly suspected[b]	2/76 (2.6%)	0/75 (0.0%)	**RR 2.97** (0.32 to 27.87)	**0 fewer per 1,000** (from 0 fewer to 0 fewer)	⊕◯◯◯ Very low	

CI: confidence interval; **RR:** risk ratio

Explanations

a. The heterogeneity between the included studies was large
b. Publication bias could not be assessed
c. Sample size less than OIS
d. The 95% confidence interval range is too large and contains invalid values
